# A Food-Grade Method for Enhancing the Levels of Low Molecular Weight Proanthocyanidins with Potentially High Intestinal Bioavailability

**DOI:** 10.3390/ijms232113557

**Published:** 2022-11-04

**Authors:** Fortuna Iannuzzo, Vincenzo Piccolo, Ettore Novellino, Elisabetta Schiano, Emanuela Salviati, Vincenzo Summa, Pietro Campiglia, Gian Carlo Tenore, Maria Maisto

**Affiliations:** 1 Department of Pharmacy, University of Naples Federico II, Via Domenico Montesano 59, 80131 Naples, Italy; 2 Faculty of Medicine and Surgery, Catholic University of the Sacred Heart, 00168 Rome, Italy; 3 Department of Pharmacy, School of Pharmacy, University of Salerno, Via Giovanni Paolo II 132, 84084 Salerno, Italy

**Keywords:** grape seeds, *Vitis vinifera*, LC/MS analysis, proanthocyanidins, antioxidant

## Abstract

Proanthocyanidins (PACs) are a group of bioactive molecules found in a variety of plants and foods. Their bioavailability depends on their molecular size, with monomers and dimers being more bioavailable than those that have a higher polymerization degree. This study aimed to develop a method to convert high-molecular-weight PACs to low-molecular-weight ones in a grape seed extract (GSE) from *Vitis vinifera* L. Therefore, GSE was subjected to alkaline treatment (ATGSE), and its difference in chemical composition, compared to GSE, was evaluated using a molecular networking (MN) approach based on results obtained from HPLC-ESI HRMS/MS characterization analysis. The network analysis mainly noted the PAC cluster with about 142 PAC compounds identified. In particular, the obtained results showed a higher content of monomeric and dimeric PACs in ATGSE compared to GSE, with 58% and 49% monomers and 31% and 24% dimers, respectively. Conversely, trimeric (9%), polymeric (4%), and galloylated PACs (14%) were more abundant in GSE than in ATGSE (6%, 1%, and 4%, respectively). Moreover, in vitro antioxidant and anti-inflammatory activities were investigated, showing the high beneficial potential of both extracts. In conclusion, ATGSE could represent an innovative natural matrix rich in bioavailable and bioaccessible PACs for nutraceutical applications with potential beneficial properties.

## 1. Introduction

Proanthocyanidins (PACs or condensed tannins) are oligomers and polymers composed of flavan-3-ol units found in many plant sources, including fruits, seeds of some plants, flowers, nuts, or barks [1]. These molecules have many biological activities, including antiviral, anti-inflammatory, radical-scavenging, antioxidant, and anticancer activities [2,3]. They are an important part of the defense mechanisms of plants due to their bitter and astringent properties useful against plant pathogens and some herbivores [2]. The most common PAs are procyanidins (PCs), which are formed by the condensation of two or monomeric flavan-3-ol units (catechin and epicatechin), and prodelfinidins (PDs), which consist of gallocatechin and/or epigallocatechin units. The monomer units of PACs can be linked by 2β→O-7 and 4β→8 linkages, known as “A”-type, or by 4β→8 linkages, known as “B”-type [4]. Finally, the linkage between C-4→C-6 is known as “C-type” linkage [5]. Generally, PACs are defined as a mixture of mainly PCs and PDs, but they may also contain propelargonidines consisting of (epi)-afzelechin subunits [6]. The bioavailability of PACs depends on the size of the molecule, with monomers and dimers absorbed and present in the blood at relatively low levels, while those with a higher degree of polymerization cannot be directly absorbed by humans [7,8]. However, larger oligomers produce smaller and more absorbable molecules, when they are metabolized by the microflora of the colon, producing metabolites with health-promoting properties [9,10]. In this regard, if polymeric PACs could be depolymerized into oligomeric ones, their bioaccessibility and bioavailability could also significantly increase [11,12]. The chemical methods investigated for the depolymerization of polymeric PACs include the use of stable composite catalysts or alcoholic solutions of mineral acids, with the addition of (+)-catechin, (−)-epicatechin, and (−)-epigallocatechin gallate as a chain breaker [13,14]. The main limitations in their use are not only the difficulty of their execution and their high costs but also, above all, the impossibility of using them for the formulation of nutraceutical products, as they are not “food-grade”. Depolymerization methods of acid-catalyzed PACs in the presence of nucleophiles, such as toluene-α-thiol or phloroglucinol, have been examined [15]. Nevertheless, these methods are difficult to use in the food industry as they produce flavan-3-ols with nucleophilic adducts that could alter the biological properties of PACs naturally present in the food matrix. Furthermore, acidic or basic methods for the depolymerization of PACs in food matrixes are described in the literature [4,16,17,18]. In particular, a study published by White et al. conducted on cranberry pomace showed that NaOH treatment resulted in an increase in low-molecular-weight procyanidins under high temperatures and short times [19]. The oligomeric PACs obtained from released hydrolysis had in vitro antioxidant and anti-inflammatory activities and have been implicated in numerous health benefits [20,21]. The best-known sources of PACs are barley, hops, apples, grapes, strawberries, cocoa, almonds, cinnamon, peanuts, and tea [22]. Among these, grape seeds are waste products of great importance in the agro-food industry because they are the most commonly produced fruit crops in the world and because they represent sources still rich in bioactive compounds [23,24]. Furthermore, PACs from grape seeds showed extensive pharmacological and therapeutic activities on health against cardiovascular diseases (CVD), diabetes mellitus, obesity, or cancer related to oxidative stress and inflammatory processes [25,26]. In light of what is stated above, the aim of this study was to develop a rapid, economical, and food-grade alkaline treatment to depolymerize grape seed extract (GSE) (*Vitis vinifera* L.) PAC polymers into lower-molecular-weight monomers and oligomers, named ATGSE (alkaline treatment of GSE). Therefore, to investigate the components present in GSE, we performed a qualitative analysis of the polyphenolic profile by UHPLC-ESI HRMS/MS. Additionally, a molecular networking (MN) approach was conducted to analyze and define the chemical composition in terms of PAC differences in both GSE and ATGSE. Finally, in vitro antioxidant and anti-inflammatory assays were carried out to evaluate the potential biological activity of GSE and ATGSE.

## 2. Results

### 2.1. UHPLC-ESI HRMS/MS and Molecular Networking (MN) Analysis of GSE and ATGSE

**Figure 1:**
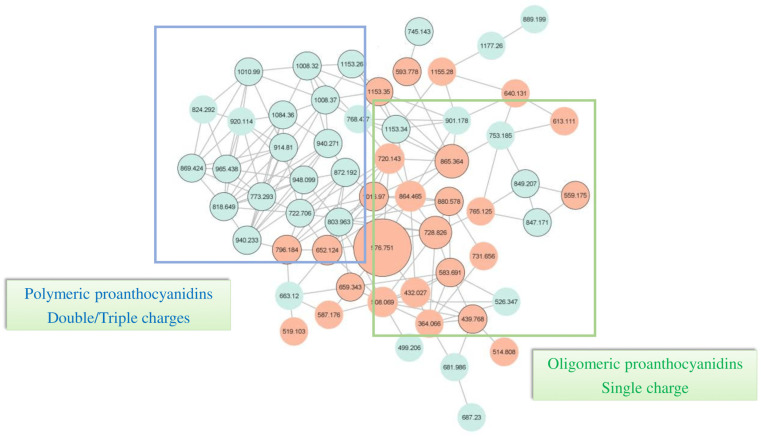
Proanthocyanidin (PAC) cluster obtained by molecular networking (MN) of grape seed extract (GSE) and its alkalinized version (ATGSE). The nodes (circles) represent a consensus MS/MS spectrum having identical precursor mass obtained from the samples, and the colors of the nodes refer to the unique property of the compound present or absent in the two samples. Blue nodes were identified in the GSE extract. Instead, red nodes represent compounds in common in the two extracts. The edges (lines) connect the nodes based on the “cosine score” (fragment match/similarity score ranging from 0.7 to 1), and the thickness of the edges reflects and measures the positive relatedness of the MS/MS spectra of the compounds within a network.

**Figure 2:**
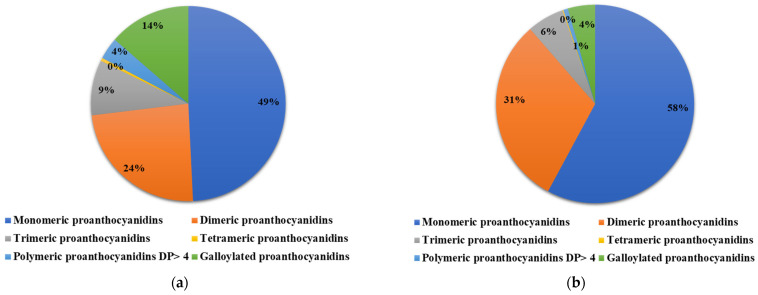
Percentage composition (area%) of proanthocyanidins (PACs) identified in (**a**) GSE sample and (**b**) ATGSE sample by UHPLC-ESI HRMS/MS analysis.

In order to evaluate the polyphenolic composition of GSE and ATGSE extracts, a UHPLC-ESI HRMS/MS analysis was performed. Compounds were identified using a molecular networking (MN) approach, which compares the MS/MS spectra of studied compounds and clusters them based on similarities between the spectra of the fragments within the dataset. MN analysis based on the compounds grouped and detected in GSE and ATGSE is shown in Supplementary Figure S1. This bioinformatic approach proved to be a high-throughput dereplication of complex matrixes to identify different groups of structural analogs. The analysis of the structural similarity demonstrated that the PAC cluster is the main cluster present in the two extracts. Therefore, the use of the network allowed the identification and characterization of 78 compounds of the PAC cluster, in addition to the identification of other compounds obtained by comparison with literature data. Supplementary Table S1 reports the UHPLC-ESI HRMS/MS analysis of PAC compounds present in GSE and ATGSE. References [27,28,29,30,31,32,33,34,35] are mentioned in the Supplementary Materials. A total of 142 PAC molecules were identified, including PACs with single, double, and triple charge, and with various degrees of polymerization. Furthermore, analogs substituted on the flavanic scaffold with galloyl groups and monomers, such as afzelechin and gallocatechin, were identified. Figure 1 reports the PAC cluster obtained from MN of GSE and ATGSE. These nodes confirmed the presence of multiple PACs, from oligomers to polymers with higher molecular weight. The result of the networking analysis showed that more polymeric PACs were present in GSE, while oligomeric PACs were predominantly present in both extracts. The difference in PAC composition was expressed as a percentage of area, as shown in Figure 2. The data showed that the content of monomeric and dimeric PACs was higher in ATGSE than in GSE, with 58% and 49% monomers and 31% and 24% dimers, respectively. The trimeric PACs were slightly higher in GSE than in ATGSE, with 9% and 6%, respectively. Furthermore, no differences in the composition of tetrameric PACs were observed in the two extracts. Finally, the GSE sample showed an elevated content of polymeric PACs with a degree of polymerization (DP) greater than 4 (4%) and galloylated PACs (14%), compared to the ATGSE sample (1% and 4%, respectively).

### 2.2. Total Phenol Content (TPC) and In Vitro Antioxidant Activity of GSE and ATGSE

The Folin-Ciocalteau assay was conducted on hydroalcoholic GSE and ATGSE extracts to evaluate their total polyphenol content (TPC). The GSE sample showed a TPC of 789 ± 0.046 mg GAE/g of extract, and ATGSE showed a TPC of 611 ± 0.004 mg GAE/g of extract (*p* < 0.001 GSE vs. ATGSE). Furthermore, the antioxidant activity of GSE and ATGSE was measured by DPPH, ABTS, and FRAP methods. The results obtained were expressed as mol TE/100 g of extract (Table 1).

**Figure 3:**
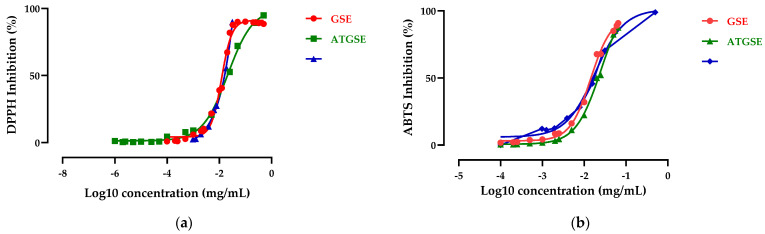
Antioxidant activity of GSE, ATGSE, and Trolox expressed as (**a**) EC_50_ of DPPH assay and (**b**) EC_50_ of ABTS assay. Values are the mean ± standard deviation (SD) of three replications.

Furthermore, the results obtained by DPPH and ABTS assays of GSE and ATGSE were also expressed as EC_50_, which is the antioxidant concentration effective in producing 50% of the maximum response, and compared with Trolox, the reference standard. As shown in Figure 3, GSE, ATGSE, and Trolox showed EC_50_ values of 0.01 mg/mL, 0.02 mg/mL, and 0.11 mg/mL for DPPH assay and of 0.01 mg/mL, 0.02 mg/mL, and 0.02 mg/mL for ABTS assay, respectively.

### 2.3. Anti-Inflammatory Activity

**Figure 4:**
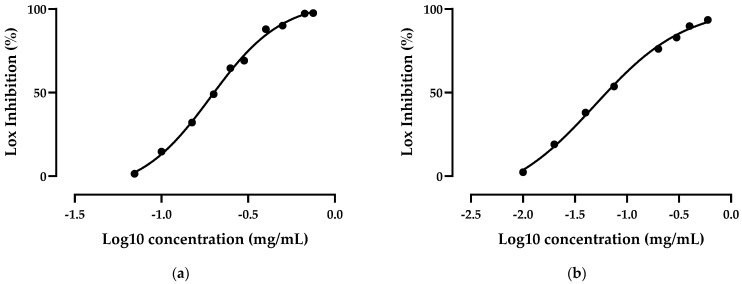
Anti-inflammatory activity evaluated by inhibition of 5-LOX activity (expressed in %) of (**a**) GSE and (**b**) ATGSE. Values are the mean ± standard deviation (SD) of three replications.

The anti-inflammatory activity of GSE and ATGSE was tested by in vitro 5-lipoxygenase (LOX) inhibition assay and cyclooxygenase (COX-1 and COX-2) inhibition assay. Figure 4 and Table 2 show the percentage of 5-LOX inhibition for GSE and ATGSE expressed as IC_50_, which is the concentration of a compound that provides a semi-maximal inhibitory effect. The results obtained revealed that GSE and ATGSE inhibited 5-LOX in a concentration-dependent manner. Zileuton was used as the reference standard, with an IC_50_ value of 0.12 ± 0.01 µg/mL. Table 1 shows the percentage of COX-1 and COX-2 inhibition for GSE and ATGSE expressed as IC_50_. The results again showed that GSE and ATGSE inhibited COX in a concentration-dependent manner. Naproxen, used as the reference standard, showed an IC_50_ value of 0.004 ± 0.001 mg/mL for COX-1 and 0.003 ± 0.015 mg/mL for COX-2.

## 3. Discussion

Proanthocyanidins (PACs) are widely present in various foods, including fruits, vegetables, and plant-based foods with potential health benefits [2,36]. However, their biological activity can change according to their composition and the degree of polymerization (DP). Many literature studies have focused on PAC oligomers with a low molecular weight (DP < 3), which are completely absorbed in the gastrointestinal tract [37]. The low bioavailability of high-molecular-weight PACs, especially polymeric ones [38], has led to the need to develop new methods that could improve this important parameter. Although several methods for depolymerization of PACs are described in the literature, only some of them are applicable in the agri-food industry. In a study published by Zhu et al. [39], it was reported that ruthenium/carbon-catalyzed depolymerization of polymeric PACs from larch bark resulted in a valuable increase in terms of oligomeric PACs. Moreover, Zhang et al. [40] have described an innovative method based on the steam explosion treatment of grape seeds aimed at depolymerizing polymeric PACs into oligomeric ones. Other authors have studied the depolymerization of polymeric PACs from grape seeds using a nucleophilic reagent [41]. However, not all methods described so far can be considered food-grade, and thus these methods are hardly suitable for industrial production. In this context, the main novelty of the present study is the development of a food-grade, as well as rapid and economical, method for large-scale production of PACs with high bioavailability. One of the main sources of PACs is grape seeds [42]. A study published by Gu et al. [43] reported that the total content of PACs in grape seeds is 35.3 mg/g of dry seed d.w. and that monomers (catechin and epicatechin) and polymers with DP > 10 were the most abundant. Although methods for depolymerization of PACs have already been described in the literature, our results showed that alkaline treatment of grape seed extract (GSE) proved to be effective in releasing PAC monomers and oligomers from the high-molecular-weight ones. In the present work, an analysis of molecular networking (MN) allowed us to compare the qualitative profile of GSE and its alkalinized version (ATGSE) in terms of the qualitative distribution of PACs. MN based on tandem mass spectrometry (MS/MS) is a recent analytical approach used to visualize and interpret the data complex from mass spectrometry analysis by grouping the MS/MS spectra based on their similarities in the fragmentation route [44]. Subsequently, the metabolites with similar fragmentation are correlated within a network, which facilitates the identification of unknown but related molecules. In the network, PACs were identified as the main cluster with 142 identified compounds, 78 of which belong to the PAC cluster, and the others were characterized by comparison with literature data. In the ESI-MS/MS spectra of the two extracts, oligomers and polymers of PAC type A and type B were observed with a degree of polymerization ranging from 2 to 10. In addition, analogs substituted on the flavanic scaffold with galloyl groups and monomers, such as afzelechin and gallocatechin, were identified. According to the study published by Monagas et al. [45], the PACs detected were essentially type B, with an abundance of about 72% compared to 8% of the corresponding type A species. The proposed mechanism to explain the observed release of low-molecular-weight PACs in ATGSE (monomeric PACs 58%, dimeric PACs 31%), under alkaline conditions and in combination with high pH and temperature, is probably due to the cleavage of the C-C interflavan bond of polymeric PACs [19]. Furthermore, the decrease in the content of galloylated PACs (4%) could be due to the hydrolysis of the bond of the galloylated esters with the release of gallic acid and the corresponding PAC [46]. The higher content of oligomeric PACs in ATGSE seems to be of great importance for potential in vivo bioactivity at the systemic level, as several studies have reported that oligomeric PACs are more bioavailable than higher oligomers [47]. Considering the nature of our food matrix, rich in antioxidant PACs, the attention was focused on the evaluation of in vitro antioxidant activity. In this regard, Folin-Ciocalteu, DPPH, ABTS, and FRAP assays were performed on GSE and ATGSE samples. The results obtained are in agreement with studies conducted on grape seed extracts (*Vitis vinifera* L.), confirming the high antioxidant power of these food waste matrixes [48]. Interestingly, it was shown that although the alkaline treatment resulted in a significant reduction in the polyphenol content in ATGSE (*p* < 0.001 vs. GSE), probably due to the different reactivity of the PACs with different molecular weights on Folin-Ciocalteu, their antioxidant potential was not significantly modified. In this respect, the results obtained showed a high antioxidant capacity of both extracts studied, comparable to the antioxidant activity of Trolox (used as the reference standard). This activity is probably attributable to the high content of proanthocyanins in grape seeds [49]. Based on this scientific evidence, we also evaluated the potential anti-inflammatory activity of our matrixes. It is well known that inflammation is a defense response of the organism, activated by various types of tissue damage (cell injury, irritation, invasion of pathogens), as well as a process to neutralize damaged and necrotic cells [50]. The development of this process relies on the involvement of numerous factors and mediators, including the release of soluble mediators (e.g., cytokines and chemokines) that recruit immune system cells to the damaged tissue, the release of arachidonic acid with the activation of the cyclooxygenase/lipoxygenase axis for the release of pro-inflammatory prostaglandins and leukotrienes, and the release of reactive oxygen species (ROS) [51,52]. In this context, we have investigated the inhibition effect of GSE and ATGSE on the three main enzymes involved in the development of an inflammatory process: COX-1, COX-2, and 5-LOX. The data obtained in this work demonstrated that the activities of lipoxygenase (5-LOX), cyclooxygenase-1 (COX-1), and cyclooxygenase-2 (COX-2) were effectively inhibited by both extracts. In particular, ATGSE showed significant inhibition of 5-LOX (*p* < 0.05 vs. GSE), while GSE showed significantly greater COX inhibitory activity (*p* < 0.05 vs. ATGSE). The high COX inhibitory activity of GSE could potentially be due to the high content of trimeric PACs, which have been shown in the literature to have potent inhibitory activity against these two inflammatory isoenzymes [53]. Furthermore, Sies et al. [54] have shown that oligomeric procyanidins can inhibit LTA4 synthase, which is the recombinant form of human 5-lipoxygenase, with a dose-dependent activity, modulating the conversion of arachidonic acid into various proinflammatory leukotrienes [55]. This could explain the greater 5-LOX inhibitory activity of ATGSE. Therefore, our research suggests that the supplementation with ATGSE and GSE could contribute to the reduction in inflammatory conditions and the prevention or treatment of oxidative stress-related diseases.

## 4. Materials and Methods

### 4.1. Reagents

All chemicals, reagents, and standards used were either analytical or LC-MS-grade reagents. Water was treated in a Milli-Q water purification system (Millipore, Bedford, MA, USA) before use. All standards and solvents for chemical analysis and in vitro studies were purchased from Sigma-Aldrich (Milan, Italy). The COX Activity Assay Kit was purchased from Cayman Chemical (Cayman Chemical Company, Ann Arbor, MI, USA) (cat No. 701050). Grape seed extract (GSE) from *Vitis vinifera* L. was purchased from MB-Med S.r.l (Turin, Italy).

### 4.2. Alkaline Treatment of GSE (ATGSE)

The alkaline treatment of GSE followed the method described by White et al. [19], with slight modifications. GSE (0.5 g) was weighed and placed in glass, screw-top tubes. Then, 20 mL of distilled water and 600 uL di NaOH 1 M were added to the tubes, and the tubes were subsequently vortexed. The tubes were then placed in a shaking bath (200 rpm) set at 45 °C for 4 h. After that, samples were removed from the water bath, frozen at −80 °C, and freeze-dried.

### 4.3. Sample Solution Preparation for UHPLC-ESI HRMS/MS Analysis

One gram of samples (GSE and ATGSE) was treated with 30 mL of MeOH/H_2_O (70:30) + 0.1% HCl 12 M with constant stirring for 30 min, at room temperature. The extracts were centrifuged at 4 °C and 6000 rpm for 10 min. The supernatants were filtered through a 0.45 µm PTFE filter and analyzed.

### 4.4. UHPLC-ESI HRMS/MS Analysis of GSE and ATGSE

UHPLC-HRMS analyses were performed on a Thermo Ultimate RS 3000 coupled online to a Q-Exactive hybrid quadrupole Orbitrap mass spectrometer (Thermo Fisher Scientific, Bremen, Germany) equipped with a heated electrospray ionization probe (HESI II) operated in negative mode. The MS was calibrated with Thermo calmix (Pierce) calibration solution. Separation was performed in RP mode using a Kinetex TM EVO C18 (150 mm × 2.1 mm; 2.6 μm) (Phenomenex, Bologna, Italy). The column temperature was set at 45 °C and the flow rate was 0.4 mL/min. The mobile phase was (A) H_2_O + 0.1% CH3COOH (*v*/*v*) and (B) ACN + 0.1% CH3COOH (*v*/*v*). The following gradient was used: 0 min, 2% B; 0.01–15 min, 25% B; 15.01–25 min, 55% B; 25.01–26 min, 98% B; 98% held for 1 min; return to 2% in 0.1 min. Four microliters was injected. Full MS (100–1500 *m*/*z*) and data-dependent MS/MS were performed at a resolution of 35,000 and 15,000 FWHM, respectively, and normalized collision energy (NCE) values of 10, 20, and 30 were used. Source parameters were: sheath gas pressure, 50 arbitrary units; auxiliary gas flow, 13 arbitrary units; spray voltage, +3.5 kV; capillary temperature, 310 °C; auxiliary gas heater temperature, 300 °C, and s-lens, 50. Two replicates of each sample were performed. Metabolite annotation was based on accurate mass measurement, MS/MS fragmentation pattern, and comparison with in silico spectra using an MS database search.

### 4.5. Molecular Networking (MN) Analysis

Mass spectra generated by HPLC-MS/MS analysis of GSE and ATGSE extracts were converted from the original “.raw” format to the “mzXML” format. The conversion was performed using MSConverter software (ProteoWizard, Palo Alto, CA USA). The data files were submitted on the GNPS platform server using the WinSCP software [56]. The molecular network data generated by GNPS software were obtained with a mass tolerance of the precursor ion (PIMT) and MS/MS fragment ion tolerance set at 0.02 Da and 0.5 Da, respectively. Consensual spectra including fewer than two similar spectra and four fragments of identical masses were removed. To reduce the complexity of the network, the spectra similarity score between clusters (cosine pairs) ranged between 0.7 and 1. MS/MS spectra were filtered by choosing the six most significant fragments to a 50 Da spectral window. The connection between clusters was provided if the individual clusters occurring in the 10 respective clusters were similar to each other, with the maximum size of a spectral family being limited to 100 clusters. The spectra in the network were searched in comparison to GNPS spectral libraries. The library spectra were filtered in the same manner as the input data. The nodes (circles) represent a consensus MS/MS spectrum having identical precursor mass obtained from the samples, and the colors of the nodes refer to the unique property of the compound present or absent in the two samples. Blue and green nodes were identified in GSE and ATGSE extracts, respectively. Instead, red nodes represent compounds in common in the two extracts. Molecular networks created were analyzed online on the GNPS platform (https://gnps.ucsd.edu/ accessed on 27 December 2021), and Cytoscape 3.9.0 was used to visualize the generated networks [57].

### 4.6. Total Phenol Content (TPC)

The total phenol content (TPC) was measured by the Folin-Ciocalteau method, using gallic acid as a standard (Sigma-Aldrich, St. Louis, MO, USA). Briefly, 0.125 mL of the sample (properly diluted with water to obtain an absorbance value within the linear range of the spectrophotometer) was added to 0.5 mL of distilled water and 0.125 mL of Folin-Ciocalteau reagent (Sigma-Aldrich, St. Louis, MO, USA). The mixture was incubated at room temperature for 6 min, and then 1.25 mL of an aqueous solution of Na_2_CO_3_ 7.5% (*w*/*v*%) was added and adjusted to 3 mL with deionized water. The absorbance was measured at 760 nm after 90 min of incubation in the dark at room temperature. Samples were analyzed in triplicate, and the results were expressed as mg of gallic acid equivalents (GAE)/g of the sample [58].

### 4.7. Antioxidant Activity

#### 4.7.1. DPPH Radical Scavenging Activity Assay

The antioxidant activity of the extracts (GSE and ATGSE) was evaluated using a 2,2-diphenyl-1-picrylhydrazyl (DPPH) (Sigma-Aldrich St. Louis, MO, USA) radical scavenging assay, as described by Maisto et al. [59]. DPPH is a stable organic nitrogen radical capable of absorbing radiation in the UV-Vis region. The reaction between DPPH and an antioxidant compound capable of donating a hydrogen atom to the radical compound leads to the decolorization of the methanolic DPPH solution due to the disappearance of the radical. A solution of 0.05 mM DPPH in methanol was prepared, and 1000 µL of this solution was mixed with 200 µL of extract in methanol at different concentrations. After mixing, the absorbance of the samples was determined spectrophotometrically at 517 nm. The percentage of DPPH inhibition was calculated according to Equation (1):

% DPPH radical scavenging activity = [(A_0_ − A_1_)/A_0_] × 100 (1)

where A_0_ is the absorbance of the control and A_1_ is the absorbance of the extracts. The 6-hydroxy-2,5,7,8-tetramethylchroman-2-carboxylic acid (Trolox) was used as the antioxidant standard, and the results were expressed in mol Trolox equivalent (TE)/100 g of the sample. Moreover, results were also expressed as EC_50_, which is the antioxidant concentration required to achieve a 50% reduction in the initial DPPH^•^ concentration. The experiment was repeated three times at each concentration.

#### 4.7.2. ABTS Radical Scavenging Activity Assay

ABTS radical cation (ABTS^+^) scavenging activity was determined according to Re et al. [60], with slight modifications. The reaction mixture was prepared with 2.5 mL of ABTS 7.0 mM solution and 44 uL of potassium persulfate 140 mM solution and left in the dark for 7 h to allow radical development. The solution was diluted with ethanol–water to achieve absorbance values of 0.7–0.75 at 734 nm. Analysis was conducted by adding 100 µL of each sample to 1 mL of the ABTS^•+^ solution. The absorbance was measured after 2.5 min of reaction at 734 nm. Ethanol was used as a blank. The scavenging effect was calculated according to Equation (2):

% ABTS radical scavenging activity = [(A_0_ − A_1_)/A_0_] × 100 (2)

where A_0_ is the absorbance of the control and A_1_ is the absorbance of the extracts. Trolox was used as the antioxidant standard. Results were expressed both as mol of TE/100 g of the sample and EC_50_, which is the antioxidant concentration required to achieve a 50% reduction in the initial ABTS^+^ concentration. The experiment was repeated three times at each concentration.

#### 4.7.3. Ferric Reducing Antioxidant Power (FRAP) Assay

The FRAP assay was conducted spectrophotometrically according to the method of Schiano et al. [61], with slight modifications. The method is based on the ability of electron-donating antioxidants to reduce the Fe^3+^ TPTZ complex (colorless complex) to Fe^2+^ tripyridyltriazine (blue-colored complex), at low pH. The FRAP solution was prepared by adding 5 mL of TPTZ (2,4,6-tris(2-pyridyl)-s-triazine) solution (10 mM) in HCl (40 mM), 5 mL of FeCl_3_ (20 mM) in water, and 50 mL of acetate buffer (0.3 M, pH 3.6). All solutions were used on the day of preparation. The mixture was preheated at 37 °C. This reagent (2.85 mL) was mixed with 0.15 mL diluted test samples at different concentrations. The absorbance was measured after 4 min at 593 nm. All determinations were performed in triplicate. A standard curve was prepared using Trolox, and the results were expressed as mol TE/100 g of sample.

### 4.8. Anti-Inflammatory Activity

#### 4.8.1. Lipoxygenase Inhibitory Activity Assay

The lipoxygenase inhibitory activity (LIA) assay was performed according to the method reported by Sharifi-Rad et al. [62], with slight modifications. Briefly, 125 µL of the extract at various concentrations was added to 125 µL of soybean lipoxidase enzyme solution (final concentration of 1250 U/mL). This mixture was incubated at 25 °C for 5 min. Then 500 µL of linoleic acid solution (358 µM) was added and the mixture was incubated again for 10 min at 25 °C. A 0.2 M borate buffer solution (pH 9) was used to dissolve all components of the assay, and 750 µL of buffer was also used to dilute the final mixture. After thorough mixing, the absorbance was measured at 234 nm. The percentage (%) inhibition was calculated according to Equation (3):

% Inhibition = [(Activity of LOX − Activity of LOX with sample)/Activity of LOX] × 100(3)

The results were expressed as IC_50_ (inhibitory concentration), which is the concentration of inhibitor at which the inhibition percentage reaches 50%. Zileuton was used as the reference anti-inflammatory compound.

#### 4.8.2. Cyclooxygenase 1 (COX-1) and Cyclooxygenase 2 (COX-2) Inhibitory Activity Assay

The cyclooxygenase 1 (COX-1) and cyclooxygenase 2 (COX-2) inhibitory activity assays were performed using a Cayman Chemical COX Colorimetric Inhibitor Screening Assay Kit (Cayman Chemical, Ann Arbor, MI, USA). The method assesses the peroxidase activity of COXs by colorimetrically monitoring the appearance of oxidized N,N,N’,N’-tetramethyl-p-phenylenediamine (TMPD) at 590 nm. Samples were divided into a positive control (100% of COX activity), containing 150 µL of 0.1 M Tris–HCl buffer (pH 8.0), 10 µL of heme, and 10 µL of enzyme, and inhibitory samples, containing 150 µL of buffer, 10 µL of heme, 10 µL of enzyme, and 10 µL of sample solution at different concentrations. Samples were incubated at 25 °C for 5 min, and then 20 µL of arachidonic acid (AA) solution and 20 µL of a colorimetric substrate solution (TMPD) were added. After 2 min of incubation at 25 °C, the absorbance at 590 nm was read. The COX-1 and COX-2 inhibitory activities were calculated as follows:

%Inhibition = [(Activity of COX − Activity of COX with sample)/Activity of COX] × 100(4)

Results were expressed as IC_50_ (inhibitory concentration), which is the concentration of inhibitor required to inhibit COX activity by 50% [63]. Naproxen was used as the reference anti-inflammatory compound.

### 4.9. Statistical Analysis

Each experiment was performed in triplicate. Values were expressed as mean ± standard deviation. Graphs were constructed and IC_50_ values were determined using GraphPad Prism 8 software. Statistical analysis of the data was performed using the Student’s *t*-test to assess significant differences between a pair of variables. *p* values below 0.05 were considered significant.

## 5. Conclusions

In conclusion, the performed food-grade alkaline treatment of GSE could represent a reliable and suitable method to increase the low-molecular-weight fraction of PACs in ATGSE. In addition, this treatment has not altered the bioactivity of the two extracts, as evidenced by their high antioxidant and anti-inflammatory activity in vitro. Therefore, although both AGSE and AGTSE have shown high antioxidant and anti-inflammatory activities, the potential supplementation with ATGSE could ensure greater intestinal bioaccessibility and systemic absorption due to its high content of oligomeric PACs.

## 6. Future Prospects

The results obtained could represent a starting point for further in vitro and in vivo studies aimed to evaluate intestinal bioaccessibility and bioavailability of the ATGSE low-molecular-weight PACs in order to assess the potential nutraceutical application of ATGSE. In addition, future studies will be aimed to evaluate the potential biological effect of the two matrixes in the prevention and treatment of inflammatory diseases.

## Figures and Tables

**Table 1:** Antioxidant activity of grape seed extract (GSE) and its alkalinized version (ATGSE) evaluated by DPPH, ABTS, and FRAP assays.

Compound	DPPH (mol TE/100 g ± SD)	ABTS (mol TE/100 g ± SD)	FRAP (mol TE/100 g ± SD)
GSE	0.39 ± 0.04	0.52 ± 0.02	0.20 ± 0.02
ATGSE	0.32 ± 0.03	0.48 ± 0.02	0.18 ± 0.02

Abbreviations: DPPH, 2,2-diphenyl-1-picrylhydrazyl; ABTS, 2,2′-azino-bis (3-ethylbenzothiazoline-6-sulfonic acid); FRAP, ferric reducing antioxidant power; TE, Trolox equivalent. Values are the mean ± standard deviation (SD) of three replications. Statistical significance was calculated by Student′s *t*-test analysis, but no significant data were found.

**Table 2:** Anti-inflammatory activity evaluated by inhibition of COX-1 and COX-2 activity (expressed in %) of GSE and ATGSE. Values are the mean ± standard deviation (SD) of three replications.

Compound	COX-1 Inhibitory Activity (IC_50_ mg/mL ± SD)	COX-2 Inhibitory Activity (IC_50_ mg/mL ± SD)	5-LOX Inhibitory Activity (IC_50_ mg/mL ± SD)
GSE	0.30 ± 0.30	0.21 ± 0.29	0.20 ± 0.01
ATGSE	1.16 ± 0.25 *	0.8 ± 0.2 *	0.05 ± 0.07 *

Values are the mean ± standard deviation (SD) of three replications. Statistical significance is calculated by Student’s *t*-test analysis: * *p* < 0.05 GSE vs. ATGSE.

## Data Availability

The data used to support the findings of this study are included in the article.

## Supplementary Materials

The following supporting information can be downloaded at: https://www.mdpi.com/article/10.3390/ijms232113557/s1. Figure S1. LC-MS/MS based Molecular Networking of compounds clustered together and detected in GSE and ATGSE. Blue and green nodes were identified in GSE and ATGSE extracts, respectively. Instead, red nodes represented compounds in common in the two extracts. The proanthocyanidin (PAC) cluster in the red box was the one identified and discussed in this article. Table S1. UHPLC-ESI HRMS/MS analysis of proanthocyanidin (PAC) compounds present in GSE and ATGSE.
