# Supplementary material for: A Food-Grade Method for Enhancing the Levels of Low Molecular Weight Proanthocyanidins with Potentially High Intestinal Bioavailability
Source: Int J Mol Sci. 2022 Nov 4;23(21):13557. doi: 10.3390/ijms232113557 (PMC9657391; doi:10.3390/ijms232113557)
Supplement: Supplementary file 1 [file ijms-23-13557-s001.zip › ijms-2000501-supplementary.pdf]

SUPPLEMENTARY MATERIALS

Figure S1. LC-MS/MS based Molecular Networking of compounds clustered together and detected in GSE and ATGSE. Blue and green nodes were identified in GSE and ATGSE extracts, respectively. Instead, red nodes represented compounds in common in the two extracts. The proanthocyanidin (PAC) cluster in the red box was the one identified and discussed in this article.

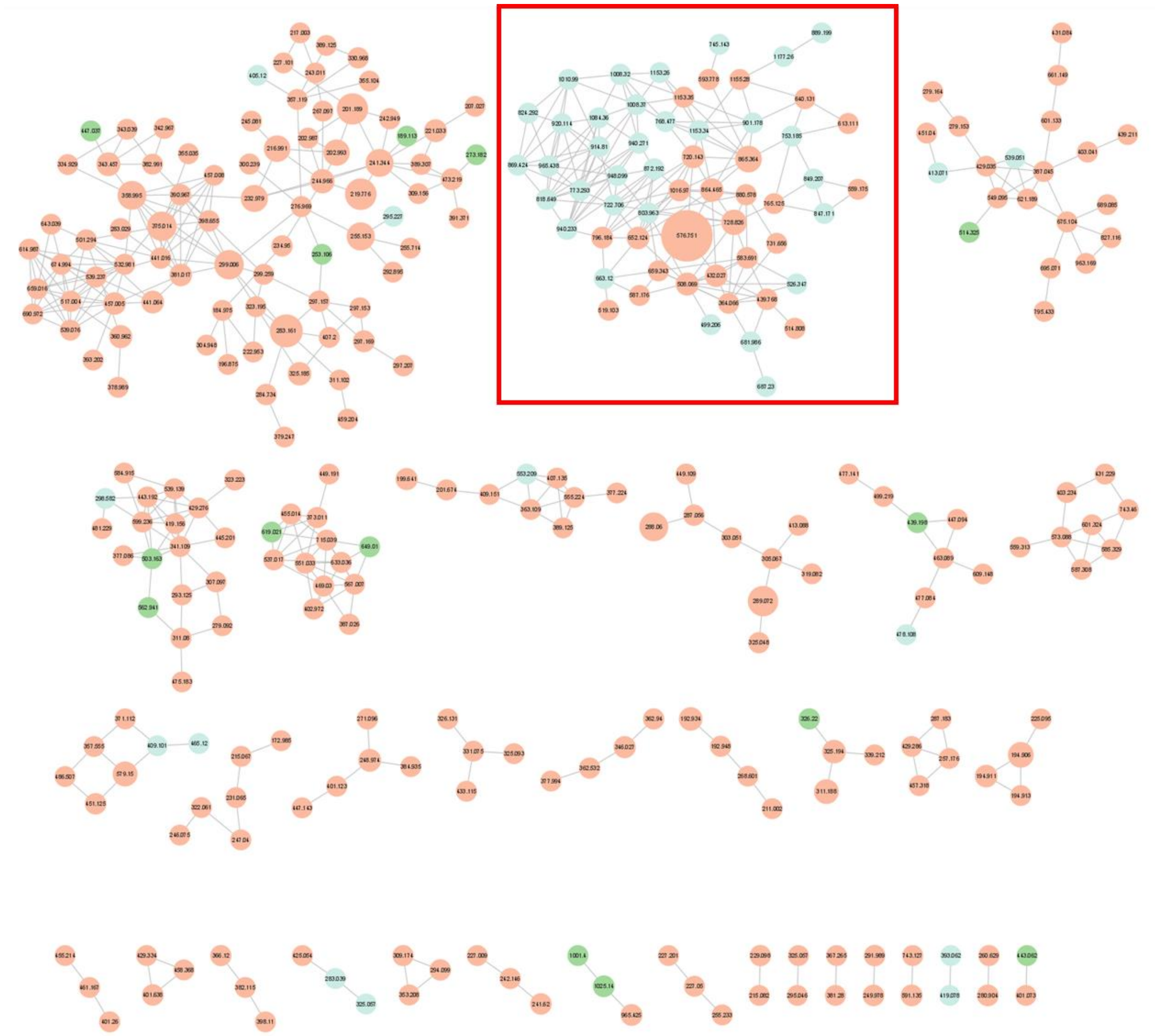

Table S1. UHPLC-ESI HRMS/MS analysis of proanthocyanidin (PAC) compounds present in GSE and ATGSE.

Compound	Charge	m/z	Formula	Error (ppm)	R_t	Fragment ions	Reference
Procyanidin trimer B-type isomer 1	$[M-H]^-$	865.2012	$C_{45}H_{37}O_{18}$	4.559	1.33	695.1379 ($-C_8H_{10}O_4$), 577.1376 ($-C_{15}H_{12}O_6$), 451.1054 ($-C_{21}H_{18}O_9$) , 425.0871 ($-C_{23}H_{20}O_9$), 407.0779 ($-C_{23}H_{22}O_{10}$), 289.0716 ($-C_{30}H_{24}O_{12}$), 287.0554 ($-C_{30}H_{26}O_{12}$), 125.0231 ($-C_{39}H_{31}O_{15}$)	[27]
Procyanidin trimer B-type isomer 2	$[M-H]^-$	865.2012	$C_{45}H_{37}O_{18}$	3.789	1.85	739.1673 ($-C_6H_6O_3$), 695.1412 ($-C_8H_{10}O_4$), 577.1353 ($-C_{15}H_{12}O_6$), 575.1199 ($-C_{15}H_{14}O_6$), 451.1039 ($-C_{21}H_{18}O_9$), 449.0876 ($-C_{21}H_{20}O_9$), 425.0884 ($-C_{23}H_{20}O_9$), 423.0718 ($-C_{23}H_{22}O_9$), 407.0772 ($-C_{23}H_{22}O_{10}$), 405.0613 ($-C_{23}H_{24}O_{10}$), 289.0719 ($-C_{30}H_{24}O_{12}$), 287.0562 ($-C_{30}H_{26}O_{12}$), 125.0233 ($-C_{39}H_{31}O_{15}$)	[27]
(Epi)gallocatechin	$[M-H]^-$	305.0667	$C_{15}H_{13}O_7$	3805	2.13	287.0562 ($-H_2O$), 179.0342 ($-C_6H_6O_3$), 137.0234 ($-C_8H_8O_4$), 125.0233 ($-C_9H_8O_4$)	[28]
(Epi)gallocatechin-(Epi)catechin isomer 1	$[M-H]^-$	593.1290	$C_{30}H_{25}O_{13}$	0.005	2.57	467.0984 ($-C_6H_6O_3$), 441.0836 ($-C_8H_8O_3$), 425.0883 ($-C_8H_8O_4$), 407.0775 ($-C_8H_{10}O_5$), 305.0674 ($-C_{15}H_{12}O_6$), 303.0512 ($-C_{15}H_{14}O_6$), 289.0720 ($-C_{15}H_{12}O_7$), 125.0233 ($-C_{24}H_{20}O_{10}$)	[29]
(Epi)catechin-(Epi)gallocatechin isomer 1	$[M-H]^-$	593.1303	$C_{30}H_{25}O_{13}$	2.264	3.03	467.0976 ($-C_6H_6O_3$), 441.0839 ($-C_8H_8O_3$), 423.0728 ($-C_8H_{10}O_4$), 305.0668 ($-C_{15}H_{12}O_6$), 289.0710 ($-C_{15}H_{12}O_7$), 287.0563 ($-C_{15}H_{14}O_7$), 269.0452 ($-C_{15}H_{16}O_8$), 125.0234 ($-C_{24}H_{20}O_{10}$)	[29]
Procyanidin trimer B-type isomer 3	$[M-H]^-$	865.2004	$C_{45}H_{37}O_{18}$	2.939	3.15	713.1530 ($-C_8H_8O_3$), 695.1426 ($-C_8H_{10}O_4$), 577.1340 ($-C_{15}H_{12}O_6$), 575.1193 ($-C_{15}H_{14}O_6$), 451.1029 ($-C_{21}H_{18}O_9$), 449.0889 ($-C_{21}H_{20}O_9$), 425.0869 ($-C_{23}H_{20}O_9$), 423.0710	[27]

						($-C_{23}H_{22}O_9$), 407.0778 ($-C_{23}H_{22}O_{10}$), 405.0618 ($-C_{23}H_{24}O_{10}$), 289.0722 ($-C_{30}H_{24}O_{12}$), 287.0563 ($-C_{30}H_{26}O_{12}$), 125.0233 ($-C_{39}H_{31}O_{15}$)	
(Epi)gallocatechin-(Epi)gallocatechin B-type linkage isomer 1	$[M-H]^-$	609.1226	$C_{30}H_{25}O_{14}$	-3.070	3.22	591.1151 ($-H_2O$) 423.0742 ($-C_8H_{10}O_5$), 303.0509 ($-C_{15}H_{14}O_7$), 285.0410 ($-C_{15}H_{16}O_8$), 125.0232 ($-C_{24}H_{20}O_{11}$)	[29]
Procyanidin pentamer B-type linkage isomer 1	$[M-2H]^{2-}$	720.1550	$C_{75}H_{60}O_{30}$	-4.098	3.70	1315.2996 ($-C_6H_5O_3$), 865.2018 ($-C_{30}H_{23}O_{12}$), 577.1329 ($-C_{45}H_{35}O_{18}$), 575.1212 ($-C_{45}H_{37}O_{18}$), 449.0874 ($-C_{51}H_{43}O_{21}$), 407.0786 ($-C_{53}H_{45}O_{22}$), 289.0720 ($-C_{60}H_{47}O_{24}$), 287.0568 ($-C_{60}H_{49}O_{24}$)	[27]
(Epi)gallocatechin-(Epi)catechin isomer 2	$[M-H]^-$	593.1300	$C_{30}H_{25}O_{13}$	1.741	3.78	467.0998 ($-C_6H_6O_3$), 441.0807 ($-C_8H_8O_3$), 425.0880 ($-C_8H_8O_4$), 407.0772 ($-C_8H_{10}O_5$), 305.0664 ($-C_{15}H_{12}O_6$), 303.0513 ($-C_{15}H_{14}O_6$), 289.0720 ($-C_{15}H_{12}O_7$), 125.0233 ($-C_{24}H_{20}O_{10}$)	[29]
(Epi)catechin *O*-hexoside isomer 1	$[M-H]^-$	451.1232	$C_{21}H_{23}O_{11}$	-0.572	3.79	289.0719 ($-C_6H_{10}O_5$), 205.0500 ($-C_{10}H_{14}O_7$), 151.0391 ($-C_{13}H_{16}O_8$), 125.0235 ($-C_{15}H_{18}O_8$)	[30]
Procyanidin dimer B-type isomer 1	$[M-H]^-$	577.1354	$C_{30}H_{25}O_{12}$	1.454	3.87	559.1238 ($-H_2O$), 451.1039 ($-C_6H_6O_3$), 425.0880 ($-C_8H_8O_3$), 407.0775 ($-C_8H_{10}O_4$), 289.0719 ($-C_{15}H_{12}O_6$), 287.0566 ($-C_{15}H_{14}O_6$), 125.0233 ($-C_{24}H_{20}O_9$)	[27]
Catechin	[M-H]-	289.0718	$C_{15}H_{13}O_6$	3.859	4.34	271.0615 ($-H_2O$), 245.0818 ($-CO_2$),179.0342 ($-C_6H_6O_2$), 125.0233 ($-C_9H_8O_3$)	[28]
(Epi)catechin-(Epi)catechin-(Epi)gallocatechin isomer 1	$[M-H]^-$	881.1949	$C_{45}H_{37}O_{19}$	2.945	4.42	577.1337 ($-C_{15}H_{12}O_7$), 451.1018 ($-C_{21}H_{18}O_{19}$), 425.0869 ($-C_{23}H_{20}O_{10}$), 407.0777 ($-C_{23}H_{22}O_{11}$), 303.0509 ($-C_{30}H_{26}O_{12}$), 289.0719 ($-C_{30}H_{24}O_{13}$), 287.0565 ($-C_{30}H_{26}O_{13}$), 125.0233 ($-C_{39}H_{32}O_{16}$)	[29]

Procyanidin trimer B-type isomer 4	$[M-H]^-$	865.2007	$C_{45}H_{37}O_{18}$	3.239	4.60	739.1646 (-$C_6H_6O_3$), 713.1530 (-$C_8H_8O_3$), 695.1365 (-$C_8H_{10}O_4$), 577.1365 (-$C_{15}H_{12}O_6$), 575.1216 (-$C_{15}H_{14}O_6$), 451.1031 (-$C_{21}H_{18}O_9$), 449.0869 (-$C_{21}H_{20}O_9$), 425.0869 (-$C_{23}H_{20}O_9$), 423.0721 (-$C_{23}H_{22}O_9$), 407.0778 (-$C_{23}H_{22}O_{10}$), 405.0604 (-$C_{23}H_{24}O_{10}$), 289.0722 (-$C_{30}H_{24}O_{12}$), 287.0564 (-$C_{30}H_{26}O_{12}$), 125.0233 (-$C_{39}H_{31}O_{15}$)	[27]
Procyanidin dimer A-type	$[M-H]^-$	575.1196	$C_{30}H_{23}O_{12}$	2.134	4.62	557.1055 (-H_2O), 449.0876 (-$C_6H_6O_3$), 423.0724 (-$C_8H_8O_3$), 407.0773 (-$C_8H_8O_4$), 289.0719 (-$C_{15}H_{10}O_6$),285.0406 (-$C_{15}H_{14}O_6$), 125.0233 (-$C_{24}H_{18}O_9$)	[31]
Procyanidin dimer B-type isomer 2	$[M-H]^-$	577.1356	$C_{30}H_{25}O_{12}$	1.766	4.65	559.1265 (-H_2O), 451.1039 (-$C_6H_6O_3$), 425.0881 (-$C_8H_8O_3$), 407.0772 (-$C_8H_{10}O_4$), 289.0718 (-$C_{15}H_{12}O_6$), 287.0563 (-$C_{15}H_{14}O_6$), 125.0233 (-$C_{24}H_{20}O_9$)	[27]
(Epi)Gallocatechin-(Epi)catechin-(Epi)catechin AB-type linkage	$[M-H]^-$	879.1812	$C_{45}H_{35}O_{19}$	4.505	4.66	709.1220 (-$C_8H_{10}O_4$), 577.1347 (-$C_{15}H_{10}O_7$), 451.1039 (-$C_{21}H_{16}O_{10}$), 449.0872 (-$C_{21}H_{18}O_{10}$), 465.0805 (-$C_{21}H_{18}O_9$), 447.0722 (-$C_{21}H_{20}O_{10}$), 421.0562 (-$C_{23}H_{22}O_{10}$), 303.0510 (-$C_{30}H_{24}O_{12}$), 289.0719 (-$C_{30}H_{22}O_{13}$), 287.0563 (-$C_{30}H_{24}O_{13}$), 178.9978 (-$C_{36}H_{28}O_{15}$), 161.0237 (-$C_{36}H_{30}O_{16}$), 125.0233 (-$C_{39}H_{30}O_{16}$)	[32]
(Epi)catechin-(Epi)gallocatechin isomer 2	$[M-H]^-$	593.1315	$C_{30}H_{25}O_{13}$	4.220	4.81	467.0982 (-$C_6H_6O_3$), 441.0831 (-$C_8H_8O_3$), 423.0721 (-$C_8H_{10}O_4$), 305.0667 (-$C_{15}H_{12}O_6$), 289.0725 (-$C_{15}H_{12}O_7$), 287.0556 (-$C_{15}H_{14}O_7$), 269.0456 (-$C_{15}H_{16}O_8$), 125.0233 (-$C_{24}H_{20}O_{10}$)	[29]
(Epi)gallocatechin-(Epi)gallocatechin B-type linkage isomer 2	$[M-H]^-$	609.1204	$C_{30}H_{25}O_{14}$	2.960	4.92	591.1160 (-H_2O), 441.0835 (-$C_8H_8O_4$), 423.0669 (-$C_8H_{10}O_5$), 303.0516	[29]

						($-C_{15}H_{14}O_7$), 285.0410 ($-C_{15}H_{16}O_8$), 125.0233 ($-C_{24}H_{20}O_{11}$)	
(Epi)catechin-(Epi)catechin-(Epi)catechin-(Epi)gallocatechin B-type linkage	[M-2H]$^{2-}$	584.1285	$C_{60}H_{48}O_{25}$	8.312	4.98	879.1683 ($-C_{15}H_{13}O_6$), 591.1125 ($-C_{30}H_{25}O_{12}$), 577.1360 ($-C_{30}H_{23}O_{13}$), 575.1193 ($-C_{30}H_{25}O_{13}$), 449.0891 ($-C_{24}H_{17}O_9$), 407.0772 ($-C_{26}H_{19}O_{10}$), 303.0508 ($-C_{33}H_{23}O_{11}$), 289.0719 ($-C_{33}H_{21}O_{12}$), 287.0563 ($-C_{33}H_{23}O_{12}$), 285.0399 ($-C_{33}H_{25}O_{12}$), 125.0233 ($-C_{55}H_{43}O_{22}$)	[29]
Procyanidin trimer B-type isomer 5	[M-H]$^-$	865.1970	$C_{45}H_{37}O_{18}$	-0.421	4.98	739.1647 ($-C_6H_6O_3$), 713.1519 ($-C_8H_8O_3$), 695.1406 ($-C_8H_{10}O_4$), 577.1354 ($-C_{15}H_{12}O_6$), 575.1194 ($-C_{15}H_{14}O_6$), 559.1254 ($-C_{15}H_{24}O_7$), 451.1035 ($-C_{21}H_{18}O_9$), 449.0879 ($-C_{21}H_{20}O_9$), 425.0880 ($-C_{23}H_{20}O_9$), 423.0715 ($-C_{23}H_{22}O_9$), 407.0770 ($-C_{23}H_{22}O_{10}$), 405.0609 ($-C_{23}H_{24}O_{10}$), 289.0719 ($-C_{30}H_{24}O_{12}$), 287.0562 ($-C_{30}H_{26}O_{12}$), 125.0233 ($-C_{39}H_{31}O_{15}$)	[27]
(Epi)catechin-(Epi)catechin-(Epi)gallocatechin isomer 2	[M-H]$^-$	881.1962	$C_{45}H_{37}O_{19}$	4.329	5.03	425.0887 ($-C_{23}H_{20}O_{10}$), 407.0779 ($-C_{23}H_{22}O_{11}$), 303.0500 ($-C_{30}H_{26}O_{12}$), 289.0725 ($-C_{30}H_{24}O_{13}$), 287.0551 ($-C_{30}H_{26}O_{13}$), 125.0235 ($-C_{39}H_{32}O_{16}$)	[29]
(Epi)Catechin-(Epi)Gallocatechin-(Epi)catechin AB-type linkage	[M-H]$^-$	879.1799	$C_{45}H_{35}O_{19}$	3.225	5.30	727.1334 ($-C_8H_8O_3$), 709.1212 ($-C_8H_{10}O_4$), 591.1157 ($-C_{15}H_{12}O_6$), 573.1049 ($-C_{15}H_{13}O_7$), 441.0834 ($-C_{23}H_{18}O_9$), 439.0663 ($-C_{23}H_{20}O_9$), 423.0735 ($-C_{23}H_{20}O_{10}$), 421.0562 ($-C_{23}H_{22}O_{10}$), 403.0458 ($-C_{23}H_{24}O_{11}$), 303.0507 ($-C_{30}H_{24}O_{12}$), 289.0720 ($-C_{30}H_{22}O_{13}$), 287.0559 ($-C_{30}H_{24}O_{13}$), 178.9964 ($-C_{36}H_{28}O_{15}$), 161.0234 ($-C_{36}H_{30}O_{16}$), 125.0232 ($-C_{39}H_{30}O_{16}$)	[32]
(Epi)catechin *O*-hexoside isomer 2	[M-H]$^-$	415.1259	$C_{21}H_{23}O_{11}$	5.325	5.40	289.0719 ($-C_6H_{10}O_5$), 271.0607	[30]

						(- $C_6H_{12}O_6$), 205.0504 (- $C_{10}H_{14}O_7$), 151.0386 (- $C_{13}H_{16}O_8$), 125.0234 (- $C_{15}H_{18}O_8$)	
(Epi)catechin-(Epi)gallocatechin A-type linkage isomer 1	[M-H]-	591.1163	$C_{30}H_{23}O_{13}$	4.169	5.41	573.1061 (- H_2O), 465.0833 (- $C_6H_6O_3$), 441.0819 (- $C_8H_6O_3$), 423.0730 (- $C_8H_8O_4$), 303.0513 (- $C_{15}H_{12}O_6$), 289.0731 (- $C_{15}H_{10}O_7$), 285.0399 (- $C_{15}H_{14}O_{17}$), 125.0233 (- $C_{24}H_{18}O_{10}$)	[29]
Procyanidin trimer B-type isomer 6	[M-H]-	865.2004	$C_{45}H_{37}O_{18}$	2.939	5.55	739.1671 (- $C_6H_6O_3$), 713.1512 (- $C_8H_8O_3$), 695.1417 (- $C_8H_{10}O_4$), 577.1357 (- $C_{15}H_{12}O_6$), 575.1198 (- $C_{15}H_{14}O_6$), 559.1269 (- $C_{15}H_{24}O_7$), 451.1040 (- $C_{21}H_{18}O_9$), 449.0880 (- $C_{21}H_{20}O_9$), 425.0881 (- $C_{23}H_{20}O_9$), 423.0717 (- $C_{23}H_{22}O_9$), 407.0771 (- $C_{23}H_{22}O_{10}$), 405.0614 (- $C_{23}H_{24}O_{10}$), 289.0719 (- $C_{30}H_{24}O_{12}$), 287.0562 (- $C_{30}H_{26}O_{12}$), 125.0233 (- $C_{39}H_{31}O_{15}$)	[27]
(Epi)afzelechin-(Epi)catechin linkage B-type isomer 1	[M-H]-	561.1415	$C_{30}H_{26}O_{11}$	1.126	5.94	435.1078 (- $C_6H_6O_3$), 409.0907 (- $C_8H_8O_3$), 407.0760 (- $C_8H_{10}O_3$), 289.0719 (- $C_{15}H_{12}O_5$), 273.0775 (- $C_{15}H_{12}O_6$), 271.0619 (- $C_{15}H_{11}O_5$), 125.0234 (- $C_{24}H_{20}O_8$)	[33]
Procyanidin tetramer B-type isomer 1	[M-H]-	1153.2637	$C_{60}H_{49}O_{24}$	1.406	5.95	1027.2397 (- $C_7H_6O_3$), 1001.2159 (- $C_8H_8O_3$), 983.2043 (- $C_8H_{10}O_4$), 865.2009 (- $C_{15}H_{12}O_6$), 863.1835 (- $C_{15}H_{14}O_6$), 739.1674 (- $C_{21}H_{18}O_9$), 713.1521 (- $C_{23}H_{20}O_9$), 577.1359 (- $C_{30}H_{24}O_{12}$), 575.1198 (- $C_{30}H_{26}O_{12}$), 451.1044 (- $C_{36}H_{30}O_{15}$), 425.0883 (- $C_{38}H_{32}O_{15}$), 407.0772 (- $C_{38}H_{34}O_{16}$), 289.0719 (- $C_{45}H_{36}O_{18}$), 287.0562 (- $C_{45}H_{38}O_{18}$), 125.0233 (- $C_{54}H_{44}O_{21}$)	[31,34]
(Epi)catechin-(Epi)catechin-(Epi)gallocatechin isomer 3	[M-H]-	881.1945	$C_{45}H_{37}O_{19}$	2.389	5.99	425.0885 (- $C_{23}H_{20}O_{10}$), 407.0767	[29]

						(-$C_{23}H_{22}O_{11}$), 303.0528 (-$C_{30}H_{26}O_{12}$), 289.0726 (-$C_{30}H_{24}O_{13}$), 287.0559 (-$C_{30}H_{26}O_{13}$), 125.0234 (-$C_{39}H_{32}O_{16}$)	
Procyanidin dimer B-type isomer 3	$[M-H]^-$	577.1356	$C_{30}H_{25}O_{12}$	1.766	6.11	559.1255 (-H_2O), 451.1037 (-$C_6H_6O_3$), 425.0881 (-$C_8H_8O_3$), 407.0772 (-$C_8H_{10}O_4$), 289.0719 (-$C_{15}H_{12}O_6$), 287.0562 (-$C_{15}H_{14}O_6$), 125.0233 (-$C_{24}H_{20}O_9$)	[27]
Epicatechin	[M-H]-	289.0718	C15H13O6	3.859	6.15	271.0610 (-H_2O), 245.0818 (-CO_2), 179.0342 (-$C_6H_6O_2$), 125.0233 (-$C_9H_8O_3$)	[28]
(Epi)catechin *O*-hexoside isomer 3	$[M-H]^-$	451.1251	$C_{21}H_{23}O_{11}$	3.618	6.26	289.0719 (-$C_6H_{10}O_5$), 205.0505 (-$C_{10}H_{14}O_7$), 151.0392 (-$C_{13}H_{16}O_8$), 125.0233 (-$C_{15}H_{18}O_8$)	[30]
Galloyl procyanidin tetramer B-type linkage isomer 1	$[M-2H]^{2-}$	652.1343	$C_{67}H_{52}O_{28}$	3.946	6.26	1179.2426 (-$C_6H_5O_3$), 1135.2598 (-$C_7H_5O_5$), 865.1956 (-$C_{22}H_{15}O_{10}$), 863.1781 (-$C_{22}H_{17}O_{10}$), 577.1347 (-$C_{37}H_{27}O_{16}$), 575.1189 (-$C_{37}H_{29}O_{16}$), 449.0880 (-$C_{43}H_{35}O_{19}$), 407.0766 (-$C_{45}H_{37}O_{20}$), 289.0719 (-$C_{52}H_{39}O_{22}$), 287.0562 (-$C_{52}H_{41}O_{22}$), 269.0445 (-$C_{52}H_{43}O_{23}$), 125.0233 (-$C_{62}H_{47}O_{25}$)	[29]
Procyanidin trimer B-type isomer 7	$[M-H]^-$	865.1996	$C_{45}H_{37}O_{18}$	2.209	6.32	739.1678 (-$C_6H_6O_3$), 713.1520 (-$C_8H_8O_3$), 695.1417 (-$C_8H_{10}O_4$), 677.1328 (-$C_8H_{12}O_5$), 577.1356 (-$C_{15}H_{12}O_6$), 575.1199 (-$C_{15}H_{14}O_6$), 559.1253 (-$C_{15}H_{24}O_7$), 557.1091 (-$C_{30}H_{26}O_7$), 451.1039 (-$C_{21}H_{18}O_9$), 449.0878 (-$C_{21}H_{20}O_9$), 425.0880 (-$C_{23}H_{20}O_9$), 423.0719 (-$C_{23}H_{22}O_9$), 407.0774 (-$C_{23}H_{22}O_{10}$), 405.0611 (-$C_{23}H_{24}O_{10}$), 289.0718 (-$C_{30}H_{24}O_{12}$), 287.0562 (-$C_{30}H_{26}O_{12}$), 125.0233 (-$C_{39}H_{31}O_{15}$)	[27]
(Epi)afzelechin-(Epi)catechin-(Epi)catechin linkage B-type isomer 1	$[M-H]^-$	849.2068	$C_{45}H_{37}O_{17}$	5.092	6.39	679.2460 (-$C_8H_{10}O_4$), 561.1423 (-$C_{15}H_{12}O_6$), 559.1234 (-$C_{15}H_{14}O_6$), 435.1065 (-$C_{24}H_{19}O_8$), 407.0779	[32]

						(- $C_{23}H_{22}O_9$), 389.0660 (- $C_{23}H_{24}O_{10}$), 289.0724 (- $C_{30}H_{24}O_{11}$), 287.0559 (- $C_{30}H_{26}O_{11}$), 273.0766 (- $C_{30}H_{24}O_{12}$), 271.0618 (- $C_{30}H_{26}O_{12}$), 125.0234 (- $C_{39}H_{32}O_{14}$)	
(Epi)gallocatechin-(Epi)gallocatechin B-type linkage isomer 3	$[M-H]^-$	609.1262	$C_{30}H_{25}O_{14}$	2.938	6.52	591.1136 (- H_2O), 423.0738 (- $C_8H_{10}O_5$), 303.0513 (- $C_{15}H_{14}O_7$), 285.0395 (- $C_{15}H_{16}O_8$), 125.0236 (- $C_{24}H_{20}O_{11}$)	[29]
O-Galloyl Procyanidin hexamer B-type linkage isomer 1	$[M-2H]^{2-}$	940.7023	$C_{97}H_{76}O_{40}$	3.468	6.54	1305.2651 (- $C_{30}H_{23}O_{12}$), 865.1904 (- $C_{52}H_{39}O_{22}$), 864.1947 (- $C_7H_4O_4$), 863.1749 (- $C_{52}H_{41}O_{22}$), 796.1590 (- $C_{15}H_{12}O_6$), 577.1367 (- $C_{67}H_{51}O_{28}$), 576.1234 (- $C_{37}H_{28}O_{16}$), 575.1205 (- $C_{67}H_{53}O_{28}$), 449.0889 (- $C_{73}H_{59}O_{31}$), 407.0765 (- $C_{75}H_{61}O_{32}$), 289.0723 (- $C_{82}H_{63}O_{34}$), 287.0561 (- $C_{82}H_{65}O_{34}$), 269.0461 (- $C_{82}H_{67}O_{35}$)	[29]
(Epi)catechin-(Epi)gallocatechin A-type linkage isomer 2	$[M-H]^-$	591.1140	$C_{30}H_{23}O_{13}$	0.244	6.56	573.1031 (- H_2O), 465.0839 (- $C_6H_6O_3$), 441.0826 (- $C_8H_6O_3$), 423.0736 (- $C_8H_8O_4$), 303.0515 (- $C_{15}H_{12}O_6$), 289.0713 (- $C_{15}H_{10}O_7$), 285.0403 (- $C_{15}H_{14}O_{17}$), 125.0234 (- $C_{24}H_{18}O_{10}$)	[29]
(Epi)catechin-(Epi)catechin *O*-hexoside B-type linkage	$[M-H]^-$	739.1827	$C_{36}H_{35}O_{17}$	-6.418	6.61	587.1391 (- $C_8H_8O_3$), 577.1337 (- $C_6H_{10}O_5$), 451.1058 (- $C_{12}H_{16}O_8$), 425.0865 (- $C_{16}H_{18}O_8$), 407.0762 (- $C_{24}H_{20}O_9$), 289.0721 (- $C_{21}H_{22}O_{11}$), 287.0558 (- $C_{21}H_{24}O_{11}$), 125.0233 (- $C_{30}H_{30}O_{14}$)	[29]
Procyanidin pentamer B-type linkage isomer 2	$[M-2H]^{2-}$	720.1590	$C_{75}H_{60}O_{30}$	1.498	6.65	1315.2932 (- $C_6H_5O_3$), 1153.2604 (- $C_{15}H_{11}O_6$), 1151.2479 (- $C_{15}H_{13}O_6$), 865.2001 (- $C_{30}H_{23}O_{12}$), 863.1839 (- $C_{30}H_{25}O_{12}$), 577.1355 (- $C_{45}H_{35}O_{18}$), 576.1245 (- $C_{15}H_{12}O_6$), 575.1201 (- $C_{45}H_{37}O_{18}$), 449.0882 (- $C_{51}H_{43}O_{21}$), 407.0774 (- $C_{53}H_{45}O_{22}$), 289.0719	[27]

						(-$C_{60}H_{47}O_{24}$), 287.0562 (-$C_{60}H_{49}O_{24}$), 269.0457 (-$C_{60}H_{51}O_{25}$)	
Epigallocatechin gallate isomer 1	$[M-H]^-$	457.0777	$C_{22}H_{17}O_{11}$	2.477	6.84	305.0668 (-$C_7H_5O_4$), 287.0567 (-$C_7H_7O_5$), 169.0134 (-$C_{15}H_{13}O_6$), 125.0234 (-$C_{16}H_{13}O_8$)	[28]
O-Galloyl-(Epi)catechin-(Epi)gallocatechin B-type linkage	$[M-H]^-$	745.1462	$C_{37}H_{29}O_{17}$	8.393	6.86	593.1268 (-$C_7H_4O_4$), 575.1175 (-$C_7H_6O_5$), 449.0873 (-$C_{13}H_{12}O_8$), 441.0821 (-$C_{15}H_{12}O_7$), 423.0722 (-$C_{15}H_{14}O_8$), 405.0622 (-$C_{15}H_{16}O_9$), 305.0672 (-$C_{22}H_{16}O_{10}$), 289.0719 (-$C_{22}H_{16}O_{11}$), 287.0568 (-$C_{22}H_{18}O_{11}$), 271.0621 (-$C_{22}H_{18}O_{12}$), 269.0453 (-$C_{22}H_20O_{12}$), 169.0135 (-$C_{20}H_{24}O_{12}$), 125.0233 (-$C_{31}H_{24}O_{14}$)	[29]
(Epi)afzelechin-(Epi)catechin linkage B-type isomer 2	$[M-H]^-$	561.1427	$C_{30}H_{26}O_{11}$	6.348	6.96	435.1092 (-$C_6H_6O_3$), 409.0923 (-$C_8H_8O_3$), 289.0719 (-$C_{15}H_{12}O_5$), 273.0771 (-$C_{15}H_{12}O_6$), 125.0233 (-$C_{24}H_20O_8$)	[33]
O-Galloyl-(Epi)catechin-(Epi)catechin-(Epi)catechin B-type linkage isomer 1	$[M-H]^-$	1017.2130	$C_{52}H_{41}O_{22}$	4.299	7.02	891.1765 (-$C_6H_6O_3$), 865.1951 (-$C_7H_4O_4$), 739.1642 (-$C_{13}H_{10}O_7$), 729.1482 (-$C_{15}H_{12}O_6$), 727 .1343 (-$C_{15}H_{14}O_6$), 721.1598 (-$C_{13}H_{12}O_8$), 577.1361 (-$C_{22}H_{16}O_{10}$), 575.1200 (-$C_{22}H_{18}O_{10}$), 559.1251 (-$C_{22}H_{18}O_{11}$), 557.1102 (-$C_{22}H_20O_{11}$), 541.1157 (-$C_{22}H_20O_{12}$), 451.1063 (-$C_{28}H_{22}O_{13}$), 433.0923 (-$C_{28}H_{24}O_{14}$), 425.0894 (-$C_{30}H_{24}O_{13}$), 423.0738 (-$C_{30}H_{26}O_{13}$), 405.0738 (-$C_{30}H_{28}O_{14}$), 387.0535 (-$C_{30}H_{30}O_{15}$), 289.0721 (-$C_{37}H_{28}O_{16}$), 287.0563 (-$C_{37}H_{30}O_{16}$), 269.0451 (-$C_{37}H_{32}O_{17}$), 169.0135 (-$C_{45}H_{36}O_{17}$), 125.0233 (-$C_{46}H_{36}O_{19}$)	[29]
Procyanidin pentamer B-type linkage isomer 3	$[M-2H]^{2-}$	720.1595	$C_{75}H_{60}O_{30}$	2.164	7.03	1315.2858 (-$C_6H_5O_3$), 1153.2686 (-$C_{15}H_{11}O_6$), 1151.2473 (-$C_{15}H_{13}O_6$), 865.1996 (-$C_{30}H_{23}O_{12}$), 863.1844	[27]

						($-C_{30}H_{25}O_{12}$), 577.1355 ($-C_{45}H_{35}O_{18}$), 576.1236 ($-C_{15}H_{12}O_6$), 575.1198 ($-C_{45}H_{37}O_{18}$), 449.0882 ($-C_{51}H_{43}O_{21}$), 407.0775 ($-C_{53}H_{45}O_{22}$), 289.0719 ($-C_{60}H_{47}O_{24}$), 287.0563 ($-C_{60}H_{49}O_{24}$), 269.0465 ($-C_{60}H_{51}O_{25}$)	
Procyanidin octamer B-type linkage isomer 1	$[M-2H]^{2-}$	1153.2638	$C_{120}H_{96}O_{48}$	2.568	7.09	865.2005 ($-C_{75}H_{59}O_{30}$), 863.1827 ($-C_{75}H_{61}O_{30}$), 577.1363 ($-C_{90}H_{71}O_{36}$), 575.1202 ($-C_{90}H_{73}O_{36}$), 449.0887 ($-C_{96}H_{79}O_{39}$), 407.0785 ($-C_{98}H_{81}O_{40}$), 289.0717 ($-C_{105}H_{83}O_{42}$), 287.0562 ($-C_{105}H_{85}O_{42}$)	[27]
(Epi)catechin-(Epi)catechin-(Epi)gallocatechin isomer 4	$[M-H]^-$	881.1956	$C_{45}H_{37}O_{19}$	3.637	7.11	577.1344 ($-C_{15}H_{12}O_7$), 425.0880 ($-C_{23}H_{20}O_{10}$), 407.0766 ($-C_{23}H_{22}O_{11}$), 303.0508 ($-C_{30}H_{26}O_{12}$), 289.0721 ($-C_{30}H_{24}O_{13}$), 287.0567 ($-C_{30}H_{26}O_{13}$), 125.0234 ($-C_{39}H_{32}O_{16}$)	[29]
O-Galloyl Procyanidin pentamer B-type linkage isomer 1	$[M-2H]^{2-}$	796.1651	$C_{82}H_{54}O_{34}$	2.134	7.11	1303.2593 ($-C_{15}H_{13}O_6$), 863.1856 ($-C_{37}H_{29}O_{16}$), 652.1348 ($-C_{15}H_{12}O_6$), 577.1374 ($-C_{52}H_{39}O_{22}$), 576.1221 ($-C_{22}H_{16}O_{10}$), 575.1192 ($-C_{52}H_{41}O_{22}$), 449.0874 ($-C_{58}H_{47}O_{25}$), 407.0775 ($-C_{60}H_{49}O_{26}$), 289.0721 ($-C_{67}H_{51}O_{28}$), 287.0565 ($-C_{67}H_{53}O_{28}$), 269.0458 ($-C_{67}H_{55}O_{29}$)	[29]
Procyanidin eptamer B-type linkage isomer 1	$[M-2H]^{2-}$	1008.7339	$C_{105}H_{84}O_{42}$	8.593	7.14	865.2001 ($-C_{60}H_{47}O_{24}$), 864.1851 ($-C_{15}H_{12}O_6$), 863.1862 ($-C_{60}H_{49}O_{24}$), 577.1368 ($-C_{75}H_{59}O_{30}$), 576.1262 ($-C_{45}H_{36}O_{18}$), 575.1204 ($-C_{75}H_{61}O_{30}$), 449.0887 ($-C_{81}H_{67}O_{33}$), 407.0781 ($-C_{83}H_{69}O_{34}$), 289.0720 ($-C_{90}H_{71}O_{36}$), 287.0563 ($-C_{90}H_{73}O_{36}$)	[27]
Galloyl-(Epi)catechin-(Epi)catechin B-type linkage isomer 1	$[M-H]^-$	729.1472	$C_{37}H_{29}O_{16}$	2.947	7.24	577.1361 ($-C_7H_4O_4$), 559.1251 ($-C_7H_6O_5$), 541.1135 ($-C_7H_8O_6$), 451.1045 ($-C_{13}H_{10}O_7$), 433.0930 ($-C_{13}H_{12}O_8$), 425.0881 ($-C_{15}H_{12}O_7$), 407.0774 ($-C_{15}H_{14}O_8$), 289.0720 ($-C_{22}H_{16}O_{10}$), 287.0563 ($-C_{22}H_{18}O_{10}$), 269.0458 (-	[29]

						$C_{22}H_{20}O_{11}$), 169.0134 (-$C_{30}H_{24}O_{11}$), 125.0233 (-$C_{31}H_{24}O_{13}$)	
(Epi)afzelechin	$[M-H]^-$	273.0771	$C_{15}H_{13}O_5$	2.899	7.40	255.0660 (-H_2O), 179.0343 (-C_6H_6O), 151.0387 (-$C_7H_6O_2$), 125.0236 (-$C_9H_8O_2$), 93.0333 (-$C_9H_8O_4$)	[27]
Procyanidin pentamer B-type linkage isomer 4	$[M-2H]^{2-}$	720.1589	$C_{75}H_{60}O_{30}$	1.414	7.42	1315.2917 (-$C_6H_5O_3$), 1153.2653 (-$C_{15}H_{11}O_6$), 1151.2482 (-$C_{15}H_{13}O_6$), 865.2023 (-$C_{30}H_{23}O_{12}$), 863.1846 (-$C_{30}H_{25}O_{12}$), 577.1354 (-$C_{45}H_{35}O_{18}$), 576.1253 (-$C_{15}H_{12}O_6$), 575.1204 (-$C_{45}H_{37}O_{18}$), 449.0880 (-$C_{51}H_{43}O_{21}$), 407.0773 (-$C_{53}H_{45}O_{22}$), 289.0719 (-$C_{60}H_{47}O_{24}$), 287.0563 (-$C_{60}H_{49}O_{24}$), 269.0446 (-$C_{60}H_{51}O_{25}$)	[27]
(Epi)catechin-(Epi)catechin-(Epi)catechin-(Epi)catechin-(Epi)catechin-(Epi)gallocatechin B-type linkage isomer 1	$[M-2H]^{2-}$	872.1936	$C_{90}H_{72}O_{37}$	7.484	7.56	879.1846 (-$C_{45}H_{37}O_{18}$), 865.1959 (-$C_{45}H_{35}O_{19}$), 863.1832 (-$C_{45}H_{37}O_{19}$), 577.1350 (-$C_{60}H_{47}O_{25}$), 576.1230 (-$C_{30}H_{22}O_{13}$), 575.1196 (-$C_{60}H_{49}O_{25}$), 449.0880 (-$C_{66}H_{55}O_{28}$), 407.0758 (-$C_{68}H_{57}O_{29}$), 303.0506 (-$C_{75}H_{61}O_{30}$), 289.0721 (-$C_{75}H_{59}O_{31}$), 287.0564 (-$C_{75}H_{61}O_{31}$), 125.0234 (-$C_{85}H_{67}O_{34}$)	[29]
(Epi)afzelechin-(Epi)catechin linkage B-type isomer 3	$[M-H]^-$	561.1382	$C_{30}H_{26}O_{11}$	4.907	7.57	435.1098 (-$C_6H_6O_3$), 425.0867 (-$C_8H_9O_2$), 407.0768 (-$C_8H_{10}O_3$), 289.0721 (-$C_{15}H_{12}O_5$), 273.0775 (-$C_{15}H_{12}O_6$), 271.0616 (-$C_{15}H_{11}O_5$), 125.0234 (-$C_{24}H_{20}O_8$)	[33]
(Epi)catechin-(Epi)gallocatechin A-type linkage isomer 3	$[M-H]^-$	591.1144	$C_{30}H_{23}O_{13}$	0.870	7.71	573.1004 (-H_2O), 465.0847 (-$C_6H_6O_3$), 441.0826 (-$C_8H_6O_3$), 423.0701 (-$C_8H_8O_4$), 303.0515 (-$C_{15}H_{12}O_6$), 289.0717 (-$C_{15}H_{10}O_7$), 285.0403 (-$C_{15}H_{14}O_{17}$), 125.0234 (-$C_{24}H_{18}O_{10}$)	[29]
(Epi)catechin-(Epi)catechin-(Epi)gallocatechin ABB linkage isomer 1	$[M-H]^-$	879.1793	$C_{45}H_{35}O_{19}$	2.282	7.83	753.1492 (-$C_6H_6O_3$), 727.1321 (-$C_8H_8O_3$), 709.1210 (-$C_8H_{10}O_4$), 591.1136 (-$C_{15}H_{12}O_6$), 577.1367 (-$C_{15}H_{10}O_7$), 575.1226 (-$C_{15}H_{12}O_7$), 573.1046 (-$C_{15}H_{14}O_7$), 555.0909 (-$C_{15}H_{16}O_8$), 465.0786 (-$C_{21}H_{18}O_9$), 441.0829 (-$C_{23}H_{18}O_9$), 423.0731 (-$C_{23}H_{20}O_{10}$), 413.0861 (-	[29]

						$C_{24}H_{18}O_{10}$), 289.0718 (-$C_{30}H_{22}O_{13}$), 287.0563 (-$C_{30}H_{24}O_{13}$), 125.0233 (-$C_{39}H_{30}O_{16}$)	
Procyanidin trimer B-type isomer 8	$[M-H]^-$	865.1998	$C_{45}H_{37}O_{18}$	1.349	7.85	739.1682 (-$C_6H_6O_3$), 713.1520 (-$C_8H_8O_3$), 695.1414 (-$C_8H_{10}O_4$), 677.1316 (-$C_8H_{12}O_5$), 577.1356 (-$C_{15}H_{12}O_6$), 575.1200 (-$C_{15}H_{14}O_6$), 559.1235 (-$C_{15}H_{24}O_7$), 557.1100 (-$C_{30}H_{26}O_7$), 451.1039 (-$C_{21}H_{18}O_9$), 449.0879 (-$C_{21}H_{20}O_9$), 425.0881 (-$C_{23}H_{20}O_9$), 423.0721 (-$C_{23}H_{22}O_9$), 407.0773 (-$C_{23}H_{22}O_{10}$), 405.0612 (-$C_{23}H_{24}O_{10}$), 289.0719 (-$C_{30}H_{24}O_{12}$), 287.0562 (-$C_{30}H_{26}O_{12}$), 125.0233 (-$C_{39}H_{31}O_{15}$)	[27]
O-Galloyl-(Epi)catechin-(Epi)catechin-(Epi)catechin B-type linkage isomer 2	$[M-H]^-$	1017.2077	$C_{52}H_{41}O_{22}$	-1.226	7.87	891.1791 (-$C_6H_6O_3$), 729.1473 (-$C_{15}H_{12}O_6$), 577.1353 (-$C_{22}H_{16}O_{10}$), 575.1180 (-$C_{22}H_{18}O_{10}$), 559.1224 (-$C_{22}H_{18}O_{11}$), 451.1049 (-$C_{28}H_{22}O_{13}$), 425.0902 (-$C_{30}H_{24}O_{13}$), 423.0724 (-$C_{30}H_{26}O_{13}$), 407.0767 (-$C_{30}H_{26}O_{14}$), 405.0604 (-$C_{30}H_{28}O_{14}$), 289.0725 (-$C_{37}H_{28}O_{16}$), 287.0563 (-$C_{37}H_{30}O_{16}$), 269.0446 (-$C_{37}H_{32}O_{17}$), 169.0136 (-$C_{45}H_{36}O_{17}$), 125.0234 (-$C_{46}H_{36}O_{19}$)	[29]
O-Galloyl Procyanidin pentamer B-type linkage isomer 2	$[M-2H]^{2-}$	796.1649	$C_{82}H_{54}O_{34}$	1.833	7.89	865.1914 (-$C_{37}H_{27}O_{16}$), 863.1806 (-$C_{37}H_{29}O_{16}$), 652.1351 (-$C_{15}H_{12}O_6$), 577.1359 (-$C_{52}H_{39}O_{22}$), 575.1196 (-$C_{52}H_{41}O_{22}$), 449.0878 (-$C_{58}H_{47}O_{25}$), 407.0773 (-$C_{60}H_{49}O_{26}$), 289.0720 (-$C_{67}H_{51}O_{28}$), 287.0562 (-$C_{67}H_{53}O_{28}$), 269.0454 (-$C_{67}H_{55}O_{29}$)	[29]
O-Galloyl Procyanidin tetramer B-type linkage	$[M-H]^-$	1305.2733	$C_{67}H_{53}O_{28}$	0.754	7.90	1153.2560 (-$C_7H_4O_4$), 1017.2159 (-$C_{15}H_{12}O_6$), 865.1931 (-$C_{22}H_{16}O_{10}$), 863.1788 (-$C_{22}H_{18}O_{10}$), 739.1690 (-$C_{28}H_{22}O_{13}$), 729.1462 (-$C_{30}H_{24}O_{12}$), 727.1279 (-$C_{30}H_{26}O_{12}$), 713.1484 (-$C_{30}H_{24}O_{13}$), 695.1437 (-$C_{30}H_{26}O_{14}$), 577.1382 (-$C_{37}H_{28}O_{16}$), 575.1196 (-$C_{37}H_{30}O_{16}$), 451.1048 (-$C_{43}H_{34}O_{19}$), 449.0887 (-$C_{43}H_{36}O_{19}$), 439.0651 (-$C_{45}H_{38}O_{19}$), 425.0886 (-$C_{45}H_{36}O_{19}$),	[29]

						423.0718 (- $C_{45}H_{38}O_{19}$), 407.0782 (- $C_{45}H_{38}O_{20}$), 405.0635 (- $C_{45}H_{40}O_{20}$), 289.0726 (- $C_{52}H_{40}O_{22}$), 287.0563 (- $C_{52}H_{42}O_{22}$), 269.0460 (- $C_{52}H_{44}O_{23}$), 125.0234 (- $C_{61}H_{48}O_{25}$)	
Epigallocatechin gallate isomer 2	$[M-H]^-$	457.0771	$C_{22}H_{17}O_{11}$	1.208	7.98	305.0671 (- $C_7H_5O_4$), 287.0563 (- $C_7H_7O_5$), 169.0134 (- $C_{15}H_{13}O_6$), 125.0233 (- $C_{16}H_{13}O_8$)	[28]
Galloyl procyanidin tetramer B-type linkage isomer 2	$[M-2H]^{2-}$	652.1336	$C_{67}H_{52}O_{28}$	2.918	8.01	1179.2415 (- $C_6H_5O_3$), 865.2000 (- $C_{22}H_{15}O_{10}$), 863.1835 (- $C_{22}H_{17}O_{10}$), 577.1359 (- $C_{37}H_{27}O_{16}$), 575.1201 (- $C_{37}H_{29}O_{16}$), 449.0881 (- $C_{43}H_{35}O_{19}$), 407.0772 (- $C_{45}H_{37}O_{20}$), 289.0718 (- $C_{52}H_{39}O_{22}$), 287.0562 (- $C_{52}H_{41}O_{22}$), 269.0445 (- $C_{52}H_{43}O_{23}$), 125.0233 (- $C_{62}H_{47}O_{25}$)	[29]
Procyanidin eptamer B-type linkage isomer 2	$[M-2H]^{2-}$	1008.7310	$C_{105}H_{84}O_{42}$	5.688	8.06	865.1953 (- $C_{60}H_{47}O_{24}$), 864.1882 (- $C_{15}H_{12}O_6$), 863.1870 (- $C_{60}H_{49}O_{24}$), 577.1364 (- $C_{75}H_{59}O_{30}$), 576.1213 (- $C_{45}H_{36}O_{18}$), 575.1207 (- $C_{75}H_{61}O_{30}$), 449.0874 (- $C_{81}H_{67}O_{33}$), 407.0774 (- $C_{83}H_{69}O_{34}$), 289.0721 (- $C_{90}H_{71}O_{36}$), 287.0562 (- $C_{90}H_{73}O_{36}$), 269.0461 (- $C_{90}H_{75}O_{37}$)	[27]
Procyanidin octamer B-type linkage isomer 2	$[M-2H]^{2-}$	1153.2635	$C_{120}H_{96}O_{48}$	2.360	8.08	865.1980 (- $C_{75}H_{59}O_{30}$), 864.1849 (- $C_{30}H_{24}O_{12}$), 863.1844 (- $C_{75}H_{61}O_{30}$), 577.1356 (- $C_{90}H_{71}O_{36}$), 575.1198 (- $C_{90}H_{73}O_{36}$), 449.0884 (- $C_{96}H_{79}O_{39}$), 407.0774 (- $C_{98}H_{81}O_{40}$), 289.0718 (- $C_{105}H_{83}O_{42}$), 287.0563 (- $C_{105}H_{85}O_{42}$), 269.0459 (- $C_{105}H_{87}O_{43}$)	[27]
Galloyl-(Epi)catechin-(Epi)catechin B-type linkage isomer 2	$[M-H]^-$	729.1468	$C_{37}H_{29}O_{16}$	2.440	8.09	577.1392 (- $C_7H_4O_4$), 541.1155 (- $C_7H_8O_6$), 451.1039 (- $C_{13}H_{10}O_7$), 441.0831 (- $C_{15}H_{12}O_6$), 433.0932 (- $C_{13}H_{12}O_8$), 425.0880 (- $C_{15}H_{12}O_7$), 407.0774 (- $C_{15}H_{14}O_8$), 289.0719 (- $C_{22}H_{16}O_{10}$), 287.0563 (- $C_{22}H_{18}O_{10}$), 269.0457 (- $C_{22}H_{20}O_{11}$), 169.0134 (- $C_{30}H_{24}O_{11}$), 125.0233 (- $C_{31}H_{24}O_{13}$)	[29]
Procyanidin dimer B-type isomer 4	$[M-H]^-$	577.1352	$C_{30}H_{25}O_{12}$	1.021	8.11	559.1257 (- H_2O), 451.1038 (- $C_6H_6O_3$), 425.0880 (- $C_8H_8O_3$), 407.0772 (- $C_8H_{10}O_4$),	[27]

						289.0719 (-$C_{15}H_{12}O_6$), 287.0562 (-$C_{15}H_{14}O_6$), 125.0233 (-$C_{24}H_{20}O_9$)	
Galloyl-(Epi)catechin-(Epi)catechin A-type linkage isomer 1	$[M-H]^-$	727.1342	$C_{37}H_{27}O_{16}$	6.682	8.14	575.1203 (-$C_7H_4O_4$), 539.0983 (-$C_7H_8O_6$), 449.0894 (-$C_{13}H_{10}O_7$), 441.0815 (-$C_{15}H_{10}O_6$), 439.0669 (-$C_{15}H_{12}O_6$), 431.0779 (-$C_{13}H_{12}O_8$), 425.0886 (-$C_{15}H_{10}O_7$), 407.0772 (-$C_{15}H_{12}O_8$), 389.0672 (-$C_{15}H_{14}O_9$), 289.0719 (-$C_{22}H_{14}O_{10}$), 287.0563 (-$C_{22}H_{16}O_{10}$), 271.0617 (-$C_{22}H_{16}O_{11}$), 269.0455 (-$C_{22}H_{18}O_{11}$), 169.0134 (-$C_{30}H_{22}O_{11}$), 125.0233 (-$C_{31}H_{22}O_{13}$)	[29]
O-Galloyl Procyanidin hexamer B-type linkage isomer 2	$[M-2H]^{2-}$	940.7009	$C_{97}H_{76}O_{40}$	1.950	8.30	1305.2760 (-$C_{30}H_{23}O_{12}$), 1303.2544 (-$C_{30}H_{25}O_{12}$), 865.1932 (-$C_{52}H_{39}O_{22}$), 863.1824 (-$C_{52}H_{41}O_{22}$), 796.1659 (-$C_{15}H_{12}O_6$), 577.1346 (-$C_{67}H_{51}O_{28}$), 576.1235 (-$C_{37}H_{28}O_{16}$), 575.1202 (-$C_{67}H_{53}O_{28}$), 449.0894 (-$C_{73}H_{59}O_{31}$), 407.0770 (-$C_{75}H_{61}O_{32}$), 289.0720 (-$C_{82}H_{63}O_{34}$), 287.0561 (-$C_{82}H_{65}O_{34}$), 269.0454 (-$C_{82}H_{67}O_{35}$)	[29]
Procyanidin tetramer B- type isomer 2	$[M-H]^-$	1153.2655	$C_{60}H_{49}O_{24}$	4.051	8.37	1027.2325 (-$C_7H_6O_3$) , 1001.2158 (-$C_8H_8O_3$) , 983.2032 (-$C_8H_{10}O_4$), 865.2002 (-$C_{15}H_{12}O_6$) , 863.1830 (-$C_{15}H_{14}O_6$) , 739.1667 (-$C_{21}H_{18}O_9$) , 713.1518 (-$C_{23}H_{20}O_9$) , 577.1355 (-$C_{30}H_{24}O_{12}$) , 575.1201 (-$C_{30}H_{26}O_{12}$), 451.1040 (-$C_{36}H_{30}O_{15}$) , 425.0879 (-$C_{38}H_{32}O_{15}$) , 407.0772 (-$C_{38}H_{34}O_{16}$) , 289.0720 (-$C_{45}H_{36}O_{18}$) , 287.0562 (-$C_{45}H_{38}O_{18}$) , 125.0233 (-$C_{54}H_{44}O_{21}$)	[31,34]
(Epi)catechin-(Epi)catechin-(Epi)catechin-(Epi)catechin-(Epi)catechin-(Epi)gallocatechin B-type linkage isomer 2	$[M-2H]^{2-}$	872.1816	$C_{90}H_{72}O_{37}$	-6.298	8.43	879.1789 (-$C_{45}H_{37}O_{18}$), 863.1887 (-$C_{45}H_{37}O_{19}$), 720.1511 (-$C_{15}H_{12}O_7$), 577.1359 (-$C_{60}H_{47}O_{25}$), 576.1225 (-$C_{30}H_{22}O_{13}$), 575.1169 (-$C_{60}H_{49}O_{25}$), 449.0862 (-$C_{66}H_{55}O_{28}$), 407.0771 (-$C_{68}H_{57}O_{29}$), 303.0509 (-$C_{75}H_{61}O_{30}$), 289.0721 (-$C_{75}H_{59}O_{31}$), 287.0562 (-$C_{75}H_{61}O_{31}$), 125.0234 (-$C_{85}H_{67}O_{34}$)	[29]

O-Galloyl-(Epi)catechin-(Epi)gallocatechin A-type linkage isomer 1	$[M-H]^-$	743.1285	$C_{37}H_{27}O_{17}$	5.000	8.43	591.1195(-$C_7H_4O_4$), 573.1058 (-$C_7H_6O_5$), 555.0934 (-$C_7H_8O_6$), 441.0810 (-$C_{15}H_{12}O_7$), 439.0671 (-$C_{15}H_{12}O_7$), 423.0724 (-$C_{15}H_{14}O_8$), 421.0567 (-$C_{15}H_{14}O_8$), 405.0625 (-$C_{15}H_{16}O_9$), 403.0455 (-$C_{15}H_{16}O_9$), 289.0712 (-$C_{22}H_{14}O_{11}$), 169.0134 (-$C_{20}H_{22}O_{12}$), 125.0234 (-$C_{31}H_{22}O_{14}$)	[29]
(Epi)catechin-(Epi)catechin-(Epi)gallocatechin ABB linkage isomer 2	$[M-H]^-$	879.1736	$C_{45}H_{35}O_{19}$	-4.178	8.46	753.1474 (-$C_6H_6O_3$), 727.1279 (-$C_8H_8O_3$), 709.1221 (-$C_8H_{10}O_4$), 591.1120 (-$C_{15}H_{12}O_6$), 575.1223 (-$C_{15}H_{12}O_7$), 573.1007 (-$C_{15}H_{14}O_7$), 465.0789 (-$C_{21}H_{18}O_9$), 441.0752 (-$C_{23}H_{18}O_9$), 423.0729 (-$C_{23}H_{20}O_{10}$), 413.0830 (-$C_{24}H_{18}O_{10}$), 289.0718 (-$C_{30}H_{22}O_{13}$), 287.0563 (-$C_{30}H_{24}O_{13}$), 125.0233 (-$C_{39}H_{30}O_{16}$)	[29]
Procyanidin pentamer B-type linkage isomer 5	$[M-2H]^{2-}$	720.1594	$C_{75}H_{60}O_{30}$	1.997	8.66	1315.2996 (-$C_6H_5O_3$), 1153.2644 (-$C_{15}H_{11}O_6$), 1151.2490 (-$C_{15}H_{13}O_6$), 865.2009 (-$C_{30}H_{23}O_{12}$), 863.1850 (-$C_{30}H_{25}O_{12}$), 577.1356 (-$C_{45}H_{35}O_{18}$), 576.1249 (-$C_{15}H_{12}O_6$), 575.1199 (-$C_{45}H_{37}O_{18}$), 449.0884 (-$C_{51}H_{43}O_{21}$), 407.0773 (-$C_{53}H_{45}O_{22}$), 289.0719 (-$C_{60}H_{47}O_{24}$), 287.0563 (-$C_{60}H_{49}O_{24}$), 269.0450 (-$C_{60}H_{51}O_{25}$)	[27]
Digalloyl procyanidin trimer B-type linkage isomer 1	$[M-2H]^{2-}$	584.1113	$C_{59}H_{44}O_{26}$	9.892	8.68	577.1360 (-$C_{29}H_{19}O_{14}$), 575.1181 (-$C_{29}H_{21}O_{14}$), 508.1024 (-$C_7H_4O_4$), 449.0898 (-$C_{35}H_{27}O_{17}$), 441.0815 (-$C_{37}H_{27}O_{16}$), 407.0780 (-$C_{37}H_{29}O_{18}$), 289.0720 (-$C_{44}H_{31}O_{20}$), 287.0720 (-$C_{44}H_{33}O_{20}$), 269.0457 (-$C_{44}H_{35}O_{21}$), 169.0133 (-$C_{52}H_{39}O_{21}$), 125.0233 (-$C_{54}H_{39}O_{23}$)	[29]
Galloyl procyanidin tetramer B-type linkage isomer 3	$[M-2H]^{2-}$	652.1326	$C_{67}H_{52}O_{28}$	1.416	8.68	1179.2493 (-$C_6H_5O_3$), 865.1957 (-$C_{22}H_{15}O_{10}$), 863.1774 (-$C_{22}H_{17}O_{10}$), 577.1362 (-$C_{37}H_{27}O_{16}$), 575.1204 (-$C_{37}H_{29}O_{16}$), 449.0882 (-$C_{43}H_{35}O_{19}$), 407.0772 (-$C_{45}H_{37}O_{20}$), 289.0719 (-$C_{52}H_{39}O_{22}$), 287.0563 (-$C_{52}H_{41}O_{22}$), 269.0453 (-$C_{52}H_{43}O_{23}$), 125.0233 (-$C_{62}H_{47}O_{25}$)	[29]

O-Galloyl Procyanidin pentamer B-type linkage isomer 3	$[M-2H]^{2-}$	796.1674	$C_{82}H_{54}O_{34}$	5.048	8.74	1305.2805 ($-C_{15}H_{11}O_6$), 1303.2512 ($-C_{15}H_{13}O_6$), 865.1991 ($-C_{37}H_{27}O_{16}$), 863.1835 ($-C_{37}H_{29}O_{16}$), 652.1334 ($-C_{15}H_{12}O_6$), 577.1368 ($-C_{52}H_{39}O_{22}$), 576.1223 ($-C_{22}H_{16}O_{10}$), 575.1195 ($-C_{52}H_{41}O_{22}$), 449.0877 ($-C_{58}H_{47}O_{25}$), 407.0774 ($-C_{60}H_{49}O_{26}$), 289.0720 ($-C_{67}H_{51}O_{28}$), 287.0564 ($-C_{67}H_{53}O_{28}$), 269.0458 ($-C_{67}H_{55}O_{29}$)	[29]
O-Galloyl-(Epi)catechin-(Epi)catechin-(Epi)catechin B-type linkage isomer 3	$[M-H]^-$	1017.2114	$C_{52}H_{41}O_{22}$	2.372	8.76	891.1774 ($-C_6H_6O_3$), 729.1465 ($-C_{15}H_{12}O_6$), 727 .1293 ($-C_{15}H_{14}O_6$), 577.1373 ($-C_{22}H_{16}O_{10}$), 575.1215 ($-C_{22}H_{18}O_{10}$), 557.1100 ($-C_{22}H_{20}O_{11}$), 451.1045 ($-C_{28}H_{22}O_{13}$), 433.0935 ($-C_{28}H_{24}O_{14}$), 425.0887 ($-C_{30}H_{24}O_{13}$), 423.0725 ($-C_{30}H_{26}O_{13}$), 407.0772 ($-C_{30}H_{26}O_{14}$), 405.0609 ($-C_{30}H_{28}O_{14}$), 289.0721 ($-C_{37}H_{28}O_{16}$), 287.0564 ($-C_{37}H_{30}O_{16}$), 269.0458 ($-C_{37}H_{32}O_{17}$), 169.0135 ($-C_{45}H_{36}O_{17}$), 125.0234 ($-C_{46}H_{36}O_{19}$)	[29]
Galloyl (Epi)catechin-(Epi)catechin-(Epi)catechin-(Epi)catechin-(Epi)catechin-(Epi)gallocatechin B-type linkage	$[M-2H]^{2-}$	948.2001	$C_{97}H_{76}O_{41}$	7.992	8.84	1131.1815 ($-C_{45}H_{35}O_{18}$), 577.1333 ($-C_{67}H_{51}O_{29}$), 576.1255 ($-C_{37}H_{28}O_{17}$), 575.1218 ($-C_{67}H_{53}O_{29}$), 449.0910 ($-C_{73}H_{59}O_{32}$), 407.0784 ($-C_{75}H_{61}O_{33}$), 303.0524 ($-C_{82}H_{65}O_{34}$), 289.0717 ($-C_{82}H_{63}O_{35}$), 287.0567 ($-C_{82}H_{65}O_{35}$)	[29]
(Epi)catechin gallate isomer 1	$[M-H]^-$	441.0829	$C_{22}H_{17}O_{10}$	2.804	8.98	331.0462 ($-C_6H_6O_2$), 303.0511 ($-C_7H_6O_3$), 289.0718 ($-C_7H_4O_4$), 271.0613 ($-C_7H_6O_5$), 169.0134 ($-C_{15}H_{12}O_5$), 125.0233 ($-C_{18}H_7O_7$)	[28]
Galloyl-(Epi)catechin-(Epi)catechin B-type linkage isomer 3	$[M-H]^-$	729.1413	$C_{37}H_{29}O_{16}$	-5.090	9.03	577.1323 ($-C_7H_4O_4$), 451.1046 ($-C_{13}H_{10}O_7$), 441.0832 ($-C_{15}H_{12}O_6$), 433.0953 ($-C_{13}H_{12}O_8$), 425.0890 ($-C_{15}H_{12}O_7$), 407.0777 ($-C_{15}H_{14}O_8$), 289.0719 ($-C_{22}H_{16}O_{10}$), 287.0556 ($-C_{22}H_{18}O_{10}$), 271.0611 ($-C_{22}H_{18}O_{12}$), 269.0466 ($-C_{22}H_{20}O_{11}$), 169.0133 ($-C_{30}H_{24}O_{11}$), 125.0233 ($-C_{31}H_{24}O_{13}$)	[29]

O-Galloyl Procyanidin pentamer B-type linkage isomer 4	$[M-2H]^{2-}$	796.1658	$C_{82}H_{54}O_{34}$	3.051	9.22	1305.2817 (-$C_{15}H_{11}O_6$), 1303.2562 (-$C_{15}H_{13}O_6$), 865.1967 (-$C_{37}H_{27}O_{16}$), 863.1857 (-$C_{37}H_{29}O_{16}$), 652.1328 (-$C_{15}H_{12}O_6$), 577.1358 (-$C_{52}H_{39}O_{22}$), 576.1236 (-$C_{22}H_{16}O_{10}$), 575.1200 (-$C_{52}H_{41}O_{22}$), 449.0878 (-$C_{58}H_{47}O_{25}$), 407.0774 (-$C_{60}H_{49}O_{26}$), 289.0720 (-$C_{67}H_{51}O_{28}$), 287.0563 (-$C_{67}H_{53}O_{28}$), 269.0457 (-$C_{67}H_{55}O_{29}$)	[29]
(Epi)afzelechin-(Epi)catechin-(Epi)catechin linkage B-type isomer 2	$[M-H]^{-}$	849.2043	$C_{45}H_{37}O_{17}$	2.148	9.23	561.1411 (-$C_{15}H_{12}O_6$), 559.1271 (-$C_{15}H_{14}O_6$), 407.0786 (-$C_{23}H_{22}O_9$), 289.0717 (-$C_{30}H_{24}O_{11}$), 287.0565 (-$C_{30}H_{26}O_{11}$), 271.0614 (-$C_{30}H_{26}O_{12}$), 125.0235 (-$C_{39}H_{32}O_{14}$)	[32]
Procyanidin octamer B-type linkage isomer 3	$[M-2H]^{2-}$	1153.2633	$C_{120}H_{96}O_{48}$	2.152	9.23	865.1973 (-$C_{75}H_{59}O_{30}$), 864.1902 (-$C_{30}H_{24}O_{12}$), 863.1861 (-$C_{75}H_{61}O_{30}$), 577.1356 (-$C_{90}H_{71}O_{36}$), 576.1217 (-$C_{60}H_{48}O_{24}$), 575.1205 (-$C_{90}H_{73}O_{36}$), 449.0890 (-$C_{96}H_{79}O_{39}$), 407.0770 (-$C_{98}H_{81}O_{40}$), 289.0721 (-$C_{105}H_{83}O_{42}$), 287.0563 (-$C_{105}H_{85}O_{42}$), 269.0455 (-$C_{105}H_{87}O_{43}$)	[27]
O-Galloyl Procyanidin hexamer B-type linkage isomer 3	$[M-2H]^{2-}$	940.6982	$C_{97}H_{76}O_{40}$	-0.879	9.25	1305.2638 (-$C_{30}H_{23}O_{12}$), 1303.2577 (-$C_{30}H_{25}O_{12}$), 865.1923 (-$C_{52}H_{39}O_{22}$), 863.1843 (-$C_{52}H_{41}O_{22}$), 796.1611 (-$C_{15}H_{12}O_6$), 577.1355 (-$C_{67}H_{51}O_{28}$), 576.1249 (-$C_{37}H_{28}O_{16}$), 575.1207 (-$C_{67}H_{53}O_{28}$), 449.0883 (-$C_{73}H_{59}O_{31}$), 407.0773 (-$C_{75}H_{61}O_{32}$), 289.0721 (-$C_{82}H_{63}O_{34}$), 287.0563 (-$C_{82}H_{65}O_{34}$), 269.0459 (-$C_{82}H_{67}O_{35}$)	[29]
Digalloyl procyanidin pentamer B-type linkage isomer 1	$[M-2H]^{2-}$	872.1750	$C_{89}H_{68}O_{38}$	7.005	9.27	865.1940 (-$C_{44}H_{31}O_{20}$), 863.1796 (-$C_{44}H_{33}O_{20}$), 796.1599 (-$C_7H_4O_4$), 729.1506 (-$C_{52}H_{39}O_{22}$), 577.1368 (-$C_{59}H_{43}O_{26}$), 576.1249 (-$C_{29}H_{20}O_{14}$), 575.1205 (-$C_{59}H_{45}O_{26}$), 449.0881 (-$C_{65}H_{51}O_{29}$), 407.0778 (-$C_{67}H_{53}O_{30}$), 289.0721 (-$C_{74}H_{55}O_{32}$), 287.0563 (-$C_{74}H_{57}O_{32}$), 269.0459 (-$C_{74}H_{59}O_{33}$)	[29]

Procyanidin hexamer B-type linkage	$[M-2H]^{2-}$	864.1895	$C_{90}H_{72}O_{36}$	-0.122	9.28	1153.2609 (-$C_{30}H_{23}O_{12}$), 1151.2482 (-$C_{30}H_{25}O_{12}$), 1135.2483 (-$C_{30}H_{25}O_{13}$), 865.2001 (-$C_{45}H_{35}O_{18}$), 863.1826 (-$C_{45}H_{37}O_{18}$), 720.1568 (-$C_{15}H_{12}O_{6}$), 577.1359 (-$C_{60}H_{47}O_{24}$), 576.1241 (-$C_{30}H_{24}O_{12}$), 575.1200 (-$C_{60}H_{49}O_{24}$), 449.0878 (-$C_{66}H_{55}O_{27}$), 407.0774 (-$C_{68}H_{57}O_{28}$), 289.0719 (-$C_{75}H_{59}O_{30}$), 287.0562 (-$C_{75}H_{61}O_{30}$), 269.0452 (-$C_{75}H_{63}O_{31}$)	[27]
(Epi)catechin-(Epi)catechin-(Epi)gallocatechin ABB linkage isomer 3	$[M-H]^{-}$	879.1769	$C_{45}H_{35}O_{19}$	-0.357	9.34	727.1299 (-$C_{8}H_{8}O_{3}$), 709.1227 (-$C_{8}H_{10}O_{4}$), 591.1163 (-$C_{15}H_{12}O_{6}$), 577.1340 (-$C_{15}H_{10}O_{7}$), 575.1220 (-$C_{15}H_{12}O_{7}$), 573.1053 (-$C_{15}H_{14}O_{7}$), 423.0715 (-$C_{23}H_{20}O_{10}$), 413.0905 (-$C_{24}H_{18}O_{10}$), 289.0721 (-$C_{30}H_{22}O_{13}$), 287.0561 (-$C_{30}H_{24}O_{13}$), 125.0233 (-$C_{39}H_{30}O_{16}$)	[29]
O-Galloyl-(Epi)catechin-(Epi)gallocatechin A-type linkage isomer 2	$[M-H]^{-}$	743.1255	$C_{37}H_{27}O_{17}$	0.896	9.41	573.1044 (-$C_{7}H_{6}O_{5}$), 455.0634 (-$C_{15}H_{12}O_{7}$), 439.0658 (-$C_{15}H_{12}O_{7}$), 421.0565 (-$C_{15}H_{14}O_{8}$), 303.0514 (-$C_{22}H_{16}O_{10}$), 289.0731 (-$C_{22}H_{14}O_{11}$), 287.0562 (-$C_{22}H_{16}O_{11}$), 285.0406 (-$C_{22}H_{18}O_{11}$), 169.0127 (-$C_{20}H_{22}O_{12}$), 125.0233 (-$C_{31}H_{22}O_{14}$)	[29]
(Epi)catechin-(Epi)gallocatechin A-type linkage isomer 4	$[M-H]^{-}$	591.1123	$C_{30}H_{23}O_{13}$	-2.648	9.49	573.1034 (-$H_{2}O$), 465.0840 (-$C_{6}H_{6}O_{3}$), 423.0727 (-$C_{8}H_{8}O_{4}$), 303.0511 (-$C_{15}H_{12}O_{6}$), 289.0719 (-$C_{15}H_{10}O_{7}$), 285.0403 (-$C_{15}H_{14}O_{17}$), 125.0234 (-$C_{24}H_{18}O_{10}$)	[29]
(Epi)afzelechin-(Epi)catechin linkage B-type isomer 4	$[M-H]^{-}$	561.1401	$C_{30}H_{26}O_{11}$	1.679	9.51	435.1064 (-$C_{6}H_{6}O_{3}$), 409.0901 (-$C_{8}H_{8}O_{3}$), 407.0785 (-$C_{8}H_{10}O_{3}$), 289.0720 (-$C_{15}H_{12}O_{5}$), 273.0770 (-$C_{15}H_{12}O_{6}$), 271.0613 (-$C_{15}H_{11}O_{5}$), 125.0281 (-$C_{24}H_{20}O_{8}$)	[33]
O-Galloyl procyanidin octamer B-type linkage isomer 1	$[M-3H]^{3-}$	819.1783	$C_{127}H_{101}O_{52}$	4.386	9.70	865.2010 (-$C_{82}H_{64}O_{34}$), 863.1857 (-$C_{82}H_{66}O_{34}$), 577.1355 (-$C_{97}H_{76}O_{40}$), 575.1202 (-$C_{97}H_{78}O_{40}$), 449.0881 (-$C_{103}H_{84}O_{43}$), 407.0772 (-$C_{105}H_{86}O_{44}$), 289.0717 (-$C_{112}H_{88}O_{46}$), 287.0564 (-$C_{112}H_{90}O_{46}$), 269.0449 (-$C_{112}H_{92}O_{47}$)	[29]

O-Galloyl Procyanidin eptamer B-type linkage isomer 1	$[M-2H]^{3-}$	722.8196	$C_{112}H_{87}O_{46}$	2.717	9.71	865.1950 (-$C_{82}H_{64}O_{34}$), 863.1845 (-$C_{82}H_{66}O_{34}$), 577.1353 (-$C_{97}H_{76}O_{40}$), 575.1203 (-$C_{97}H_{78}O_{40}$), 449.0874 (-$C_{103}H_{84}O_{43}$), 407.0770 (-$C_{105}H_{86}O_{44}$), 289.0719 (-$C_{112}H_{88}O_{46}$), 287.0562 (-$C_{112}H_{90}O_{46}$), 269.0458 (-$C_{112}H_{92}O_{47}$)	[29]
Trigalloyl procyanidin tetramer B-type linkage isomer 1	$[M-2H]^{2-}$	804.1475	$C_{81}H_{60}O_{36}$	5.962	9.78	1169.2173 (-$C_{22}H_{15}O_{10}$), 1167.2109 (-$C_{22}H_{17}O_{10}$), 729.1448 (-$C_{44}H_{31}O_{20}$), 728.1385 (-$C_{7}H_{4}O_{4}$), 727.1270 (-$C_{44}H_{33}O_{20}$), 577.1387 (-$C_{51}H_{35}O_{24}$), 575.1208 (-$C_{51}H_{37}O_{24}$), 449.0878 (-$C_{57}H_{43}O_{27}$), 441.0818 (-$C_{59}H_{43}O_{26}$), 407.0766 (-$C_{59}H_{45}O_{28}$), 289.0723 (-$C_{66}H_{47}O_{30}$), 287.0563 (-$C_{66}H_{49}O_{30}$), 269.0451 (-$C_{66}H_{51}O_{31}$), 169.0135 (-$C_{74}H_{55}O_{31}$), 125.0234 (-$C_{76}H_{55}O_{33}$)	[29]
Digalloyl procyanidin trimer B-type linkage isomer 2	$[M-2H]^{2-}$	584.1073	$C_{59}H_{44}O_{26}$	3.095	9.84	881.1605 (-$C_{15}H_{11}O_{6}$), 577.1368 (-$C_{29}H_{19}O_{14}$), 575.1204 (-$C_{29}H_{21}O_{14}$), 508.0984 (-$C_{7}H_{4}O_{4}$), 449.0883 (-$C_{35}H_{27}O_{17}$), 441.0832 (-$C_{37}H_{27}O_{16}$), 407.0775 (-$C_{37}H_{29}O_{18}$), 289.0720 (-$C_{44}H_{31}O_{20}$), 287.0564 (-$C_{44}H_{33}O_{20}$), 269.0458 (-$C_{44}H_{35}O_{21}$), 169.0134 (-$C_{52}H_{39}O_{21}$), 125.0233 (-$C_{54}H_{39}O_{23}$)	[29]
(Epi)catechin gallate isomer 2	$[M-H]^{-}$	441.0826	$C_{22}H_{17}O_{10}$	2.169	9.88	331.0454 (-$C_{6}H_{6}O_{2}$), 303.0510 (-$C_{7}H_{6}O_{3}$), 289.0719 (-$C_{7}H_{4}O_{4}$), 271.0610 (-$C_{7}H_{6}O_{5}$), 169.0134 (-$C_{15}H_{12}O_{5}$), 125.0233 (-$C_{18}H_{7}O_{7}$)	[28]
Digalloyl procyanidin pentamer B-type linkage isomer 2	$[M-2H]^{2-}$	872.1754	$C_{89}H_{68}O_{38}$	7.498	9.95	1017.2163 (-$C_{37}H_{27}O_{16}$), 796.1569 (-$C_{7}H_{4}O_{4}$), 729.1451 (-$C_{52}H_{39}O_{22}$), 577.1370 (-$C_{59}H_{43}O_{26}$), 576.1243 (-$C_{29}H_{20}O_{14}$), 575.1195 (-$C_{59}H_{45}O_{26}$), 449.0890 (-$C_{65}H_{51}O_{29}$), 407.0770 (-$C_{67}H_{53}O_{30}$), 289.0721 (-$C_{74}H_{55}O_{32}$), 287.0564 (-$C_{74}H_{57}O_{32}$), 269.0454 (-$C_{74}H_{59}O_{33}$)	[29]
Digalloyl (Epi)catechin-(Epi)catechin B-type linkage	$[M-2H]^{2-}$	440.0744	$C_{44}H_{32}O_{20}$	1.436	10.05	711.1374 (-$C_{7}H_{5}O_{5}$), 449.0877 (-$C_{20}H_{15}O_{11}$), 407.0774 (-$C_{22}H_{17}O_{12}$), 364.0699 (-$C_{7}H_{4}O_{4}$), 289.0720 (-$C_{29}H_{19}O_{14}$), 287.0564 (-	[29]

						$C_{29}H_{21}O_{14}$), 269.0458 (-$C_{29}H_{23}O_{15}$), 169.0134 (-$C_{37}H_{27}O_{15}$), 125.0233 (-$C_{39}H_{27}O_{17}$)	
Procyanidin octamer B-type linkage isomer 4	$[M-2H]^{2-}$	1153.2639	$C_{120}H_{96}O_{48}$	2.881	10.05	1008.1849 (-$C_{15}H_{12}O_6$), 865.2004 (-$C_{75}H_{59}O_{30}$), 864.1948 (-$C_{30}H_{24}O_{12}$), 863.1862 (-$C_{75}H_{61}O_{30}$), 577.1352 (-$C_{90}H_{71}O_{36}$), 576.1220 (-$C_{60}H_{48}O_{24}$), 575.1198 (-$C_{90}H_{73}O_{36}$), 449.0873 (-$C_{96}H_{79}O_{39}$), 407.0768 (-$C_{98}H_{81}O_{40}$), 289.0722 (-$C_{105}H_{83}O_{42}$), 287.0564 (-$C_{105}H_{85}O_{42}$), 269.0466 (-$C_{105}H_{87}O_{43}$)	[27]
Digallate (Epi)catechin-(Epi)catechin B-type linkage	$[M-H]^-$	881.1589	$C_{44}H_{33}O_{20}$	2.669	10.06	729.1482 (-$C_7H_4O_4$), 711.1340 (-$C_7H_6O_5$), 603.1172 (-$C_{13}H_{10}O_7$), 577.1380 (-$C_{14}H_8O_8$), 559.1274 (-$C_{14}H_{10}O_9$), 541.1144 (-$C_{14}H_{12}O_{10}$), 451.1057 (-$C_{20}H_{14}O_{11}$), 433.0934 (-$C_{20}H_{16}O_{12}$), 425.0875 (-$C_{22}H_{16}O_{11}$), 407.0774 (-$C_{22}H_{18}O_{12}$), 389.0681 (-$C_{22}H_{20}O_{13}$), 289.0720 (-$C_{29}H_{20}O_{14}$), 287.0564 (-$C_{29}H_{22}O_{14}$), 271.0616 (-$C_{29}H_{22}O_{15}$), 269.0457 (-$C_{29}H_{24}O_{15}$), 169.0134 (-$C_{37}H_{28}O_{15}$), 125.0233 (-$C_{38}H_{28}O_{17}$)	[29]
Trigalloyl procyanidin octamer B-type linkage	$[M-3H]^{3-}$	930.1877	$C_{141}H_{107}O_{60}$	9.015	10.10	864.1874 (-$C_{51}H_{35}O_{24}$), 577.1361 (-$C_{111}H_{82}O_{48}$), 575.1218 (-$C_{111}H_{84}O_{48}$), 449.0882 (-$C_{117}H_{90}O_{51}$), 441.0847 (-$C_{119}H_{90}O_{50}$), 407.0786 (-$C_{119}H_{92}O_{52}$), 289.0720 (-$C_{126}H_{94}O_{54}$), 287.0565 (-$C_{126}H_{96}O_{54}$), 269.0462 (-$C_{126}H_{98}O_{55}$)	[29]
(Epi)afzelechin-(Epi)catechin linkage B-type isomer 5	$[M-H]^-$	561.1411	$C_{30}H_{26}O_{11}$	3.514	10.22	435.1067 (-$C_6H_6O_3$), 425.0861 (-$C_8H_9O_2$), 409.0935 (-$C_8H_8O_3$), 289.0725 (-$C_{15}H_{12}O_5$), 273.0768 (-$C_{15}H_{12}O_6$), 271.0609 (-$C_{15}H_{11}O_5$), 125.0234 (-$C_{24}H_{20}O_8$)	[33]
Procyanidin dimer B-type isomer 5	$[M-H]^-$	577.1354	$C_{30}H_{25}O_{12}$	1.454	10.22	559.1273 (-H_2O), 451.1038 (-$C_6H_6O_3$), 425.0882 (-$C_8H_8O_3$), 407.0773 (-$C_8H_{10}O_4$), 289.0719 (-$C_{15}H_{12}O_6$), 287.0563 (-$C_{15}H_{14}O_6$), 125.0233 (-$C_{24}H_{20}O_9$)	[27]

O-Galloyl-(Epi)catechin-(Epi)catechin-(Epi)catechin) B-type linkage isomer 4	$[M-H]^-$	1017.2136	$C_{52}H_{41}O_{22}$	4.535	10.34	891.1794 (-$C_6H_6O_3$), 729.1474 (-$C_{15}H_{12}O_6$), 575.1193 (-$C_{22}H_{18}O_{10}$), 557.1069 (-$C_{22}H_{20}O_{11}$), 541.1155 (-$C_{22}H_{20}O_{12}$), 451.1039 (-$C_{28}H_{22}O_{13}$), 433.0954 (-$C_{28}H_{24}O_{14}$), 425.0879 (-$C_{30}H_{24}O_{13}$), 423.0721 (-$C_{30}H_{26}O_{13}$), 407.0771 (-$C_{30}H_{26}O_{14}$), 405.0621 (-$C_{30}H_{28}O_{14}$), 289.0719 (-$C_{37}H_{28}O_{16}$), 287.0564 (-$C_{37}H_{30}O_{16}$), 271.0605 (-$C_{37}H_{30}O_{17}$), 269.0446 (-$C_{37}H_{32}O_{17}$), 169.0134 (-$C_{45}H_{36}O_{17}$), 125.0234 (-$C_{46}H_{36}O_{19}$)	[29]
Digalloyl procyanidin eptamer B-type linkage	$[M-3H]^{3-}$	773.4949	$C_{119}H_{91}O_{50}$	8.887	10.38	865.2014 (-$C_{74}H_{54}O_{32}$), 846.1640 (-$C_{29}H_{19}O_{14}$), 722.4777 (-$C_7H_4O_4$), 577.1332 (-$C_{89}H_{66}O_{38}$), 576.1224 (-$C_{59}H_{43}O_{26}$), 575.1207 (-$C_{89}H_{68}O_{38}$), 449.0888 (-$C_{95}H_{74}O_{41}$), 407.0759 (-$C_{97}H_{76}O_{42}$), 289.0721 (-$C_{104}H_{78}O_{44}$), 287.0563 (-$C_{104}H_{90}O_{44}$), 269.0452 (-$C_{104}H_{82}O_{45}$)	[29]
(Epi)catechin-(Epi)catechin-(Epi)afzelechin AB-type linkage	$[M-H]^-$	847.1937	$C_{45}H_{35}O_{17}$	4.774	10.48	695.1470 (-$C_8H_8O_3$), 577.1416 (-$C_{15}H_{10}O_5$), 557.1088 (-$C_{15}H_{14}O_6$), 425.0902 (-$C_{23}H_{18}O_8$), 407.0770 (-$C_{23}H_{20}O_9$), 289.0713 (-$C_{30}H_{22}O_{11}$), 287.0566 (-$C_{30}H_{24}O_{11}$), 269.0454 (-$C_{30}H_{26}O_{12}$), 125.0234 (-$C_{39}H_{30}O_{14}$)	
(Epi)catechin-(Epi)catechin-(Epi)catechin-(Epi)catechin-(Epi)catechin-(Epi)catechin-(Epi)catechin-(Epi)catechin-(Epi)gallocatechin B-type linkage isomer 1	$[M-3H]^{3-}$	869.1913	$C_{135}H_{107}O_{55}$	6.866	10.54	865.1903 (-$C_{90}H_{70}O_{37}$), 864.1877 (-$C_{45}H_{35}O_{19}$), 863.1887 (-$C_{90}H_{72}O_{37}$), 591.1143 (-$C_{105}H_{84}O_{42}$), 577.1374 (-$C_{105}H_{82}O_{43}$), 575.1216 (-$C_{105}H_{84}O_{43}$), 467.0970 (-$C_{111}H_{88}O_{45}$), 449.0900 (-$C_{111}H_{90}O_{46}$), 441.0821 (-$C_{113}H_{90}O_{45}$), 407.0782 (-$C_{113}H_{92}O_{47}$), 303.0513 (-$C_{120}H_{96}O_{48}$), 289.0722 (-$C_{120}H_{94}O_{49}$), 287.0564 (-$C_{120}H_{96}O_{49}$), 285.0403 (-$C_{120}H_{98}O_{49}$), 269.0463 (-$C_{120}H_{98}O_{50}$)	[29]
Galloyl-(Epi)catechin-(Epi)catechin B-type linkage isomer 4	$[M-H]^-$	729.1475	$C_{37}H_{29}O_{16}$	3.359	10.60	577.1367 (-$C_7H_4O_4$), 559.1242 (-$C_7H_6O_5$), 541.1143 (-$C_7H_8O_6$), 451.1056 (-$C_{13}H_{10}O_7$), 433.0928 (-$C_{13}H_{12}O_8$), 425.0887 (-$C_{15}H_{12}O_7$),	[29]

						407.0773 (-$C_{15}H_{14}O_8$), 289.0719 (-$C_{22}H_{16}O_{10}$), 287.0565 (-$C_{22}H_{18}O_{10}$), 271.0620 (-$C_{22}H_{18}O_{12}$), 269.0461 (-$C_{22}H_{20}O_{11}$), 169.0134 (-$C_{30}H_{24}O_{11}$), 125.0233 (-$C_{31}H_{24}O_{13}$)	
Procyanidin eptamer B-type linkage isomer 3	$[M-2H]^{2-}$	1008.7337	$C_{105}H_{84}O_{42}$	8.405	10.66	865.1964 (-$C_{60}H_{47}O_{24}$), 864.1894 (-$C_{15}H_{12}O_6$), 863.1843 (-$C_{60}H_{49}O_{24}$), 577.1338 (-$C_{75}H_{59}O_{30}$), 576.1224 (-$C_{45}H_{36}O_{18}$), 575.1203 (-$C_{75}H_{61}O_{30}$), 449.0881 (-$C_{81}H_{67}O_{33}$), 407.0770 (-$C_{83}H_{69}O_{34}$), 289.0719 (-$C_{90}H_{71}O_{36}$), 287.0563 (-$C_{90}H_{73}O_{36}$)	[27]
Digalloyl procyanidin pentamer B-type linkage isomer 3	$[M-2H]^{2-}$	872.1744	$C_{89}H_{68}O_{38}$	8.375	10.71	1017.2058 (-$C_{37}H_{27}O_{16}$), 865.2001 (-$C_{44}H_{31}O_{20}$), 863.1843 (-$C_{44}H_{33}O_{20}$), 796.1594 (-$C_7H_4O_4$), 729.1429 (-$C_{52}H_{39}O_{22}$), 577.1368 (-$C_{59}H_{43}O_{26}$), 576.1241 (-$C_{29}H_{20}O_{14}$), 575.1201 (-$C_{59}H_{45}O_{26}$), 449.0895 (-$C_{65}H_{51}O_{29}$), 407.0775 (-$C_{67}H_{53}O_{30}$), 289.0720 (-$C_{74}H_{55}O_{32}$), 287.0563 (-$C_{74}H_{57}O_{32}$), 269.0458 (-$C_{74}H_{59}O_{33}$)	[29]
Trigalloyl procyanidin pentamer B-type linkage	$[M-2H]^{2-}$	948.1808	$C_{96}H_{72}O_{42}$	6.782	10.71	872.1625 (-$C_7H_4O_4$), 577.1359 (-$C_{96}H_{70}O_{42}$), 576.1261 (-$C_{66}H_{47}O_{30}$), 575.1202 (-$C_{96}H_{72}O_{42}$), 449.0871 (-$C_{102}H_{78}O_{45}$), 407.0770 (-$C_{104}H_{80}O_{46}$), 289.0725 (-$C_{111}H_{82}O_{49}$), 287.0564 (-$C_{111}H_{84}O_{49}$), 269.0459 (-$C_{111}H_{86}O_{50}$)	[29]
Procyanidin trimer B-type isomer 9	$[M-H]^-$	865.1956	$C_{45}H_{37}O_{18}$	-1.881	10.74	577.1349 (-$C_{15}H_{12}O_6$), 575.1188 (-$C_{15}H_{14}O_6$), 451.1035 (-$C_{21}H_{18}O_9$), 449.0885 (-$C_{21}H_{20}O_9$), 425.0867 (-$C_{23}H_{20}O_9$), 423.0733 (-$C_{23}H_{22}O_9$), 407.0770 (-$C_{23}H_{22}O_{10}$), 405.0609 (-$C_{23}H_{24}O_{10}$), 289.0720 (-$C_{30}H_{24}O_{12}$), 287.0564 (-$C_{30}H_{26}O_{12}$), 125.0234 (-$C_{39}H_{31}O_{15}$)	[27]
O-Galloyl Procyanidin nonamer B-type linkage isomer 1	$[M-3H]^{3-}$	915.1989	$C_{142}H_{113}O_{58}$	3.249	10.78	1303.2610 (-$C_{75}H_{62}O_{30}$), 1151.2389 (-$C_{82}H_{66}O_{34}$), 865.1985 (-$C_{97}H_{76}O_{40}$), 864.1826 (-$C_7H_5O_4$), 864.1826 (-$C_{52}H_{41}O_{22}$), 863.1846 (-$C_{97}H_{78}O_{40}$), 720.1542 (-$C_{67}H_{53}O_{28}$), 577.1345 (-$C_{112}H_{88}O_{46}$), 575.1201 (-	[29]

						$C_{112}H_{90}O_{46}$), 449.0893 (-$C_{118}H_{96}O_{49}$), 407.0787 (-$C_{120}H_{98}O_{50}$), 289.0721 (-$C_{127}H_{100}O_{52}$), 287.0562 (-$C_{127}H_{102}O_{52}$), 269.0445 (-$C_{127}H_{104}O_{53}$)	
O-Galloyl Procyanidin eptamer B-type linkage isomer 2	$[M-2H]^{3-}$	722.8196	$C_{112}H_{87}O_{46}$	-3.666	11.01	865.1907 (-$C_{67}H_{50}O_{28}$), 864.1947 (-$C_{22}H_{15}O_{10}$), 863.1754 (-$C_{67}H_{52}O_{28}$), 577.1349 (-$C_{97}H_{76}O_{40}$), 576.1264 (-$C_{52}H_{29}O_{22}$), 575.1216 (-$C_{97}H_{78}O_{40}$), 449.0881 (-$C_{103}H_{84}O_{43}$), 407.0770 (-$C_{105}H_{86}O_{44}$), 289.0720 (-$C_{112}H_{88}O_{46}$), 287.0564 (-$C_{112}H_{90}O_{46}$), 269.0462 (-$C_{112}H_{92}O_{47}$)	[29]
Digalloyl procyanidin trimer B-type linkage isomer 3	$[M-2H]^{2-}$	584.1075	$C_{59}H_{44}O_{26}$	3.403	11.05	577.1293 (-$C_{29}H_{19}O_{14}$), 575.1221 (-$C_{29}H_{21}O_{14}$), 508.1018 (-$C_{7}H_{4}O_{4}$), 449.0897 (-$C_{35}H_{27}O_{17}$), 441.0821 (-$C_{37}H_{27}O_{16}$), 407.0782 (-$C_{37}H_{29}O_{18}$), 289.0721 (-$C_{44}H_{31}O_{20}$), 287.0563 (-$C_{44}H_{33}O_{20}$), 269.0457 (-$C_{44}H_{35}O_{21}$), 169.0135 (-$C_{52}H_{39}O_{21}$), 125.0234 (-$C_{54}H_{39}O_{23}$)	[29]
O-Galloyl procyanidin octamer B-type linkage isomer 2	$[M-3H]^{3-}$	819.1696	$C_{127}H_{101}O_{52}$	-6.332	11.09	1152.2498 (-$C_{7}H_{5}O_{4}$), 865.1938 (-$C_{97}H_{76}O_{40}$), 863.1829 (-$C_{97}H_{78}O_{40}$), 577.1364 (-$C_{97}H_{76}O_{40}$), 575.1222 (-$C_{97}H_{78}O_{40}$), 449.0861 (-$C_{103}H_{84}O_{43}$), 407.0785 (-$C_{105}H_{86}O_{44}$), 289.0719 (-$C_{112}H_{88}O_{46}$), 287.0562 (-$C_{112}H_{90}O_{46}$), 269.0461 (-$C_{112}H_{92}O_{47}$)	[29]
O-Galloyl Procyanidin hexamer B-type linkage isomer 4	$[M-2H]^{2-}$	940.7008	$C_{97}H_{76}O_{40}$	1.916	11.36	1305.2745 (-$C_{30}H_{23}O_{12}$), 1303.2551 (-$C_{30}H_{25}O_{12}$) 863.1827 (-$C_{52}H_{41}O_{22}$), 796.1663 (-$C_{15}H_{12}O_{6}$), 577.1394 (-$C_{67}H_{51}O_{28}$), 576.1237 (-$C_{37}H_{28}O_{16}$), 575.1199 (-$C_{67}H_{53}O_{28}$), 449.0879 (-$C_{73}H_{59}O_{31}$), 407.0775 (-$C_{75}H_{61}O_{32}$), 289.0717 (-$C_{82}H_{63}O_{34}$), 287.0564 (-$C_{82}H_{65}O_{34}$), 269.0455 (-$C_{82}H_{67}O_{35}$)	[29]
(Epi)catechin-(Epi)catechin-(Epi)catechin-(Epi)catechin-(Epi)catechin-(Epi)catechin-(Epi)catechin-(Epi)catechin-	$[M-3H]^{3-}$	869.1787	$C_{135}H_{107}O_{55}$	-7.596	11.38	1153.2574 (-$C_{75}H_{58}O_{31}$), 865.1953 (-$C_{90}H_{70}O_{37}$), 864.1877 (-$C_{45}H_{35}O_{19}$), 863.1802 (-$C_{90}H_{72}O_{37}$), 577.1380 (-$C_{105}H_{82}O_{43}$), 575.1204 (-$C_{105}H_{84}O_{43}$), 449.0885 (-	[29]

(Epi)gallocatechin B-type linkage isomer 2						$C_{111}H_{90}O_{46}$), 441.0773 (-$C_{113}H_{90}O_{45}$), 407.0750 (-$C_{113}H_{92}O_{47}$), 303.0511 (-$C_{120}H_{96}O_{48}$), 289.0722 (-$C_{120}H_{94}O_{49}$), 287.0562 (-$C_{120}H_{96}O_{49}$), 285.0404 (-$C_{120}H_{98}O_{49}$), 269.0467 (-$C_{120}H_{98}O_{50}$)	
Trigalloyl procyanidin tetramer B-type linkage isomer 2	$[M-2H]^{2-}$	804.1484	$C_{81}H_{60}O_{36}$	7.181	11.63	881.1562 (-$C_{37}H_{27}O_{16}$), 729.1483 (-$C_{44}H_{31}O_{20}$), 728.1401 (-$C_{7}H_{4}O_{4}$), 727.1401 (-$C_{44}H_{33}O_{20}$), 652.1304 (-$C_{14}H_{8}O_{8}$), 577.1293 (-$C_{51}H_{35}O_{24}$), 575.1191 (-$C_{51}H_{37}O_{24}$), 449.0881 (-$C_{57}H_{43}O_{27}$), 441.0818 (-$C_{59}H_{43}O_{26}$), 407.0768 (-$C_{59}H_{45}O_{28}$), 289.0722 (-$C_{66}H_{47}O_{30}$), 287.0563 (-$C_{66}H_{49}O_{30}$), 269.0457 (-$C_{66}H_{51}O_{31}$), 169.0134 (-$C_{74}H_{55}O_{31}$), 125.0234 (-$C_{76}H_{55}O_{33}$)	[29]
Digalloyl procyanidin octamer B-type linkage	$[M-3H]^{3-}$	869.8469	$C_{134}H_{103}O_{56}$	2.061	11.94	1152.2460 (-$C_{14}H_{7}O_{8}$), 863.1775 (-$C_{89}H_{68}O_{38}$), 818.8378 (-$C_{7}H_{4}O_{4}$), 577.1349 (-$C_{104}H_{78}O_{44}$), 576.1218 (-$C_{74}H_{55}O_{32}$), 575.1218 (-$C_{104}H_{80}O_{44}$), 449.0897 (-$C_{110}H_{86}O_{47}$), 407.0765 (-$C_{112}H_{88}O_{48}$), 289.0721 (-$C_{119}H_{90}O_{50}$), 287.0721 (-$C_{119}H_{92}O_{50}$), 285.0415 (-$C_{119}H_{94}O_{50}$), 269.0464 (-$C_{119}H_{94}O_{51}$)	[29]
O-Galloyl procyanidin octamer B-type linkage isomer 3	$[M-3H]^{3-}$	819.1705	$C_{127}H_{101}O_{52}$	-5.222	11.96	1153.2711 (-$C_{67}H_{52}O_{28}$), 865.2057 (-$C_{82}H_{64}O_{34}$), 863.1875 (-$C_{82}H_{66}O_{34}$), 577.1321 (-$C_{97}H_{76}O_{40}$), 575.1207 (-$C_{97}H_{78}O_{40}$), 449.0870 (-$C_{103}H_{84}O_{43}$), 407.0770 (-$C_{105}H_{86}O_{44}$), 289.0719 (-$C_{112}H_{88}O_{46}$), 287.0565 (-$C_{112}H_{90}O_{46}$), 269.0458 (-$C_{112}H_{92}O_{47}$)	[29]
Procyanidin trimer B-type isomer 10	$[M-H]^{-}$	865.2017	$C_{45}H_{37}O_{18}$	4.279	12.01	577.1280 (-$C_{15}H_{12}O_{6}$), 449.0901 (-$C_{21}H_{20}O_{9}$), 407.0771 (-$C_{23}H_{22}O_{10}$), 289.0723 (-$C_{30}H_{24}O_{12}$), 287.0565 (-$C_{30}H_{26}O_{12}$), 125.0233 (-$C_{39}H_{31}O_{15}$)	[27]
(Epi)catechin-(Epi)catechin-(Epi)catechin-(Epi)catechin-(Epi)catechin-	$[M-3H]^{3-}$	965.5389	$C_{150}H_{119}O_{61}$	-3.628	12.07	864.1818 (-$C_{60}H_{47}O_{25}$), 863.1837 (-$C_{105}H_{84}O_{43}$), 577.1330 (-$C_{120}H_{94}O_{49}$),	[29]

(Epi)catechin-(Epi)catechin-(Epi)catechin-(Epi)catechin-(Epi)catechin-(Epi)gallocatechin B-type linkage						576.1245 (-$C_{90}H_{71}O_{37}$), 575.1207 (-$C_{120}H_{96}O_{49}$), 449.0891 (-$C_{126}H_{102}O_{52}$), 441.0853 (-$C_{128}H_{102}O_{51}$), 407.0771 (-$C_{128}H_{104}O_{53}$), 303.0511 (-$C_{135}H_{108}O_{54}$), 289.0720 (-$C_{135}H_{106}O_{55}$), 287.0560 (-$C_{135}H_{108}O_{55}$), 285.0425 (-$C_{135}H_{110}O_{55}$), 269.0450 (-$C_{135}H_{110}O_{56}$)	
O-Galloyl Procyanidin decamer B-type linkage	$[M-3H]^{3-}$	1010.8876	$C_{157}H_{121}O_{64}$	-9.078	12.20	1303.2552 (-$C_{90}H_{70}O_{36}$), 1151.2513 (-$C_{97}H_{74}O_{40}$), 863.1835 (-$C_{18}H_{16}O_{10}$), 577.1340 (-$C_{127}H_{96}O_{52}$), 575.1202 (-$C_{127}H_{98}O_{52}$), 449.0876 (-$C_{133}H_{104}O_{55}$), 407.0767 (-$C_{135}H_{106}O_{56}$), 289.0726 (-$C_{142}H_{108}O_{58}$), 287.0565 (-$C_{142}H_{110}O_{58}$), 269.0466 (-$C_{142}H_{112}O_{59}$)	[29]
O-Galloyl-(Epi)catechin-(Epi)catechin-(Epi)catechin B-type linkage isomer 5	$[M-H]^{-}$	1017.2135	$C_{52}H_{41}O_{22}$	4.476	12.23	891.1757 (-$C_6H_6O_3$), 729.1478 (-$C_{15}H_{12}O_6$), 577.1303 (-$C_{22}H_{16}O_{10}$), 575.1201 (-$C_{22}H_{18}O_{10}$), 451.1046 (-$C_{28}H_{22}O_{13}$), 433.0941 (-$C_{28}H_{24}O_{14}$), 407.0777 (-$C_{30}H_{26}O_{14}$), 405.0586 (-$C_{30}H_{28}O_{14}$), 289.0720 (-$C_{37}H_{28}O_{16}$), 287.0562 (-$C_{37}H_{30}O_{16}$), 269.0454 (-$C_{37}H_{32}O_{17}$), 169.0133 (-$C_{45}H_{36}O_{17}$), 125.0234 (-$C_{46}H_{36}O_{19}$)	[29]
Trigalloyl procyanidin trimer B-type linkage	$[M-2H]^{2-}$	660.1140	$C_{66}H_{48}O_{30}$	4.512	12.33	729.1481 (-$C_{29}H_{19}O_{14}$), 584.1064 (-$C_7H_4O_4$), 575.1143 (-$C_{36}H_{25}O_{18}$), 449.0884 (-$C_{42}H_{31}O_{21}$), 407.0785 (-$C_{44}H_{33}O_{22}$), 289.0723 (-$C_{51}H_{35}O_{24}$), 287.0564 (-$C_{51}H_{37}O_{24}$), 269.0439 (-$C_{51}H_{39}O_{25}$), 169.0134 (-$C_{59}H_{43}O_{25}$), 125.0233 (-$C_{61}H_{41}O_{27}$)	[29]
O-Galloyl Procyanidin nonamer B-type linkage isomer 2	$[M-3H]^{3-}$	915.2023	$C_{142}H_{113}O_{58}$	7.051	12.35	865.1936 (-$C_{97}H_{76}O_{40}$), 863.1855 (-$C_{97}H_{78}O_{40}$), 577.1354 (-$C_{112}H_{88}O_{46}$), 575.1206 (-$C_{112}H_{90}O_{46}$), 449.0875 (-$C_{118}H_{96}O_{49}$), 407.0771 (-$C_{120}H_{98}O_{50}$), 289.0721 (-$C_{127}H_{100}O_{52}$), 287.0563 (-$C_{127}H_{102}O_{52}$), 269.0464 (-$C_{127}H_{104}O_{53}$)	[29]

Digalloyl procyanidin nonamer B-type linkage	$[M-3H]^{3-}$	965.5285	$C_{149}H_{115}O_{62}$	-1.817	12.40	914.8511 (-$C_7H_4O_4$), 865.1961 (-$C_{104}H_{78}O_{44}$), 864.1833 (-$C_{14}H_8O_8$), 577.1349 (-$C_{119}H_{90}O_{50}$), 575.1204 (-$C_{119}H_{92}O_{50}$), 449.0895 (-$C_{125}H_{98}O_{53}$), 407.0786 (-$C_{127}H_{100}O_{54}$), 289.0719 (-$C_{134}H_{102}O_{56}$), 287.0565 (-$C_{134}H_{104}O_{56}$), 269.0470 (-$C_{134}H_{106}O_{57}$)	[29]
O-Galloyl Procyanidin hexamer B-type linkage isomer 5	$[M-2H]^{2-}$	940.7065	$C_{97}H_{76}O_{40}$	8.006	12.42	1305.2733 (-$C_{30}H_{23}O_{12}$), 863.1790 (-$C_{52}H_{41}O_{22}$), 577.1319 (-$C_{67}H_{51}O_{28}$), 576.1246 (-$C_{37}H_{28}O_{16}$), 575.1220 (-$C_{67}H_{53}O_{28}$), 449.0874 (-$C_{73}H_{59}O_{31}$), 407.0775 (-$C_{75}H_{61}O_{32}$), 289.0721 (-$C_{82}H_{63}O_{34}$), 287.0564 (-$C_{82}H_{65}O_{34}$), 269.0457 (-$C_{82}H_{67}O_{35}$)	[29]
(Epi)catechin *O*-coumarate isomer 1	$[M-H]^-$	453.1088	$C_{24}H_{19}O_8$	-4.950	12.47	341.0670 (-C_6H_6O), 289.0709 (-$C_9H_7O_2$), 125.0233 (-$C_{18}H_{14}O_5$)	[30]
Galloyl-(Epi)catechin-(Epi)catechin B-type linkage isomer 5	$[M-H]^-$	729.1458	$C_{37}H_{29}O_{16}$	1.013	12.52	541.1138 (-$C_7H_8O_6$), 451.1042 (-$C_{13}H_{10}O_7$), 433.0936 (-$C_{13}H_{12}O_8$), 425.0887 (-$C_{15}H_{12}O_7$), 407.0776 (-$C_{15}H_{14}O_8$), 289.0721 (-$C_{22}H_{16}O_{10}$), 287.0563 (-$C_{22}H_{18}O_{10}$), 271.0616 (-$C_{22}H_{18}O_{12}$), 269.0461 (-$C_{22}H_{20}O_{11}$), 169.0135 (-$C_{30}H_{24}O_{11}$), 125.0234 (-$C_{31}H_{24}O_{13}$)	
(Epi)catechin-(Epi)catechin-(Epi)catechin-(Epi)catechin-(Epi)catechin-(Epi)catechin-(Epi)catechin-(Epi)catechin-(Epi)gallocatechin B-type linkage isomer 3	$[M-3H]^{3-}$	869.1905	$C_{135}H_{107}O_{55}$	5.957	12.53	863.1798 (-$C_{90}H_{72}O_{37}$), 577.1362 (-$C_{105}H_{82}O_{43}$), 575.1206 (-$C_{105}H_{84}O_{43}$), 449.0882 (-$C_{111}H_{90}O_{46}$), 441.0789 (-$C_{113}H_{90}O_{45}$), 407.0763 (-$C_{113}H_{92}O_{47}$), 303.0503 (-$C_{120}H_{96}O_{48}$), 289.0721 (-$C_{120}H_{94}O_{49}$), 287.0565 (-$C_{120}H_{96}O_{49}$), 285.0401 (-$C_{120}H_{98}O_{49}$), 269.0457 (-$C_{120}H_{98}O_{50}$)	[29]
Galloyl-(Epi)catechin-(Epi)catechin A-type linkage isomer 2	$[M-H]^-$	727.1348	$C_{37}H_{27}O_{16}$	7.521	12.53	575.1212 (-$C_7H_4O_4$), 557.1100 (-$C_7H_6O_5$), 539.1041 (-$C_7H_8O_6$), 449.0873 (-$C_{13}H_{10}O_7$), 431.0801 (-$C_{13}H_{12}O_8$), 407.0775 (-$C_{15}H_{12}O_8$), 389.0672 (-$C_{15}H_{14}O_9$), 289.0719 (-$C_{22}H_{14}O_{10}$), 287.0563 (-$C_{22}H_{16}O_{10}$), 271.0617 (-$C_{22}H_{16}O_{11}$), 269.0455 (-$C_{22}H_{18}O_{11}$), 169.0134 (-$C_{30}H_{22}O_{11}$), 125.0233 (-$C_{31}H_{22}O_{13}$)	[29]

(Epi)catechin-(Epi)catechin-(Epi)catechin AB-type linkage	$[M-H]^-$	863.1827	$C_{45}H_{35}O_{18}$	1.100	12.62	737.1558 (-$C_6H_6O_3$), 711.1359 (-$C_8H_8O_3$), 577.1359 (-$C_{15}H_{10}O_6$), 575.1192 (-$C_{15}H_{12}O_6$), 573.1013 (-$C_{15}H_{14}O_6$), 449.0872 (-$C_{21}H_{18}O_9$), 425.0855 (-$C_{23}H_{18}O_9$), 407.0755 (-$C_{23}H_{20}O_{10}$), 289.0722 (-$C_{30}H_{22}O_{12}$), 287.0562 (-$C_{30}H_{24}O_{12}$), 269.0451 (-$C_{30}H_{26}O_{13}$)	[27]
Galloyl-(Epi)catechin-(Epi)catechin-(Epi)catechin AB-type linkage	$[M-H]^-$	863.2051	$C_{52}H_{39}O_{22}$	9.999	13.09	863.1784 (-$C_7H_4O_4$), 575.1194 (-$C_{22}H_{16}O_{10}$), 449.0875 (-$C_{28}H_{22}O_{13}$), 407.0768 (-$C_{30}H_{24}O_{14}$), 289.0720 (-$C_{37}H_{26}O_{16}$), 287.0564 (-$C_{37}H_{28}O_{16}$), 269.0458 (-$C_{37}H_{30}O_{17}$)	[29]
(Epi)catechin-(Epi)afzelechin-(Epi)catechin AB-type linkage isomer 1	$[M-H]^-$	847.1932	$C_{45}H_{35}O_{17}$	7.512	13.20	559.1289 (-$C_{15}H_{12}O_6$), 433.0930 (-$C_{21}H_{18}O_9$), 407.0790 (-$C_{23}H_{20}O9$), 289.0717 (-$C_{30}H_{22}O_{11}$), 287.0543 (-$C_{30}H_{24}O_{11}$), 269.0454 (-$C_{30}H_{26}O_{12}$), 125.0235 (-$C_{39}H_{30}O_{14}$)	[29]
(Epi)catechin ethyl dimer isomer 1	$[M-H]^-$	605.1677	$C_{32}H_{29}O_{12}$	3.813	13.38	453.1169 (-$C_8H_8O_3$), 315.0879 (-$C_{15}H_{15}O_6$), 289.0722 (-$C_{17}H_{16}O_6$), 125.0235 (-$C_{26}H_{24}O_9$)	[35]
(Epi)catechin-(Epi)afzelechin A-type linkage isomer 1	$[M-H]^-$	559.1250	$C_{30}H_{23}O_{11}$	2.704	13.78	433.0931 (-$C_6H_6O_3$), 407.0775 (-$C_8H_8O_3$), 289.0720 (-$C_{15}H_{10}O_5$), 269.0458 (-$C_{15}H_{14}O_6$), 125.0231 (-$C_{24}H_{18}O_8$)	[29]
(Epi)catechin *O*-coumarate isomer 2	$[M-H]^-$	453.1101	$C_{24}H_{19}O_8$	-0.048	14.26	341.0669 (-C_6H_6O), 289.0721 (-$C_9H_7O_2$), 165.0186 (-$C_{16}H_{14}O_4$), 125.0231 (-$C_{18}H_{14}O_5$)	[30]
(Epi)catechin-(Epi)afzelechin-(Epi)catechin AB-type linkage isomer 2	$[M-H]^-$	847.1944	$C_{45}H_{35}O_{17}$	8.881	14.64	559.1257 (-$C_{15}H_{12}O_6$), 433.0933 (-$C_{21}H_{18}O_9$), 407.0786 (-$C_{23}H_{20}O_9$), 289.0721 (-$C_{30}H_{22}O_{11}$), 287.0561 (-$C_{30}H_{24}O_{11}$), 269.0450 (-$C_{30}H_{26}O_{12}$), 125.0234 (-$C_{39}H_{30}O_{14}$)	[29]
(Epi)catechin ethyl dimer isomer 2	$[M-H]^-$	605.1677	$C_{32}H_{29}O_{12}$	3.813	14.96	453.1224 (-$C_8H_8O_3$), 315.0877 (-$C_{15}H_{15}O_6$), 289.0721 (-$C_{17}H_{16}O_6$), 125.0232 (-$C_{26}H_{24}O_9$)	[35]
(Epi)catechin-(Epi)afzelechin A-type linkage isomer 2	$[M-H]^-$	559.1266	$C_{30}H_{23}O_{11}$	5.655	15.29	433.0932 (-$C_6H_6O_3$), 407.0785 (-$C_8H_8O_3$), 289.0718 (-$C_{15}H_{10}O_5$), 269.0457 (-$C_{15}H_{14}O_6$), 125.0228 (-$C_{24}H_{18}O_8$)	[29]
(Epi)catechin *O*-coumarate isomer 3	$[M-H]^-$	453.1053	$C_{24}H_{19}O_8$	-4.950	16.42	289.0720 (-$C_9H_7O_2$), 165.0189 (-$C_{16}H_{14}O_4$), 125.0231 (-$C_{18}H_{14}O_5$)	[30]
